# A multi-residue method for trace analysis of pesticides in soils with special emphasis on rigorous quality control

**DOI:** 10.1007/s00216-023-04872-8

**Published:** 2023-08-08

**Authors:** Andrea Rösch, Felix E. Wettstein, Daniel Wächter, Vanessa Reininger, Reto G. Meuli, Thomas D. Bucheli

**Affiliations:** 1 Environmental Analytics, Agroscope, 8046 Zurich, Switzerland; 2 Soil Quality and Soil Use, Agroscope, 8046 Zurich, Switzerland

**Keywords:** Soil monitoring, Pesticide exposure, Method development and validation, Quality assurance and quality control, Plant protection product

## Abstract

A multi-residue trace analytical method is presented to accurately quantify 146 currently used pesticides in (agricultural) soils with varying soil properties. Pesticides were extracted using an optimized quick, easy, cheap, effective, rugged, and safe (QuEChERS) approach and chemical analysis was carried out by liquid chromatography coupled to tandem mass spectrometry (triple quadrupole). Quantification was based on matrix-matched internal standards calibration, using 95 isotopically labeled analyte analogues. In contrast to the common approach of method validation using soils freshly spiked with analytes shortly before the extraction, our method is additionally validated via an in-house prepared partly aged soil, which contains all target pesticides and via agricultural field soils with native pesticide residues. The developed method is highly sensitive (median method limit of quantification: 0.2 ng/g), precise (e.g., median intra-day and inter-day method precision both ~ 4% based on field soils), and true ((i) quantified pesticide concentrations of the partly aged soil remained stable during 6 months, were close to the initially spiked nominal concentration of 10 ng/g, and thus can be used to review trueness in the future; (ii) median freshly spiked relative recovery: 103%; and (iii) participation in a ring trial: median *z*-scores close to one (good to satisfactory result)). Its application to selected Swiss (agricultural) soils revealed the presence of in total 77 different pesticides with sum concentrations up to 500 ng/g. The method is now in use for routine soil monitoring as part of the *Swiss Action Plan for Risk Reduction and Sustainable Use of Plant Protection Products*.

**Graphical abstract:**

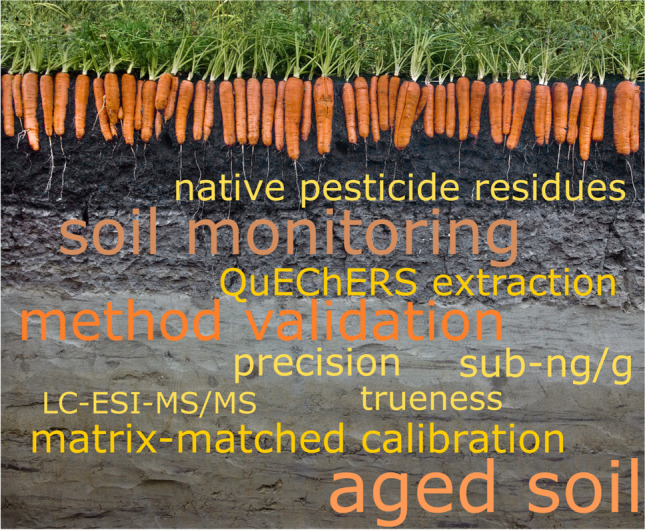

**Supplementary information:**

The online version contains supplementary material available at 10.1007/s00216-023-04872-8.

## Introduction

Pesticides are applied on agricultural fields to fight or prevent pests, diseases, and weeds in order to maintain crop yields. While the worldwide amount of used pesticides has remained stable over the last decade with  ~ 2.6 million tons per year [1], the toxicity of applied pesticides towards invertebrates and plants has increased considerably [2]. This implies that the potential environmental impact of pesticides is not solely consumption-based, but depends on the specific highly variable toxicity of individual pesticides towards non-target organisms. Whereas risk-based environmental quality standards (EQS) are available for selected pesticides to protect surface water bodies in the European Union [3] and Switzerland [4], hardly any related EQS (soil protection values) for pesticides exist worldwide to protect soil life [5], although soil in agriculturally influenced areas is a primary recipient of pesticides. Additionally, information on soil contamination with pesticides is relatively scarce compared to available monitoring data from surface water bodies. Nevertheless, recent studies performed in Switzerland [6, 7] and throughout Europe [8–16] show the ubiquitous appearance of pesticides in agricultural soils even in untreated areas such as organically managed fields or extensively managed grassland sites [17, 18].

To assess short- and long-term ecotoxicological effects on soil life, a terrestrial risk assessment is needed. Therefore, within the *Swiss Action Plan for Risk Reduction and Sustainable Use of Plant Protection Products (AP PPP)* [19], adopted by the Federal Council in 2017 with the overall goal to reduce the risk associated with pesticides by 50% and to promote alternatives to chemical pest control, one measure focuses on the development of a long-term monitoring of pesticide residues in agricultural soils. This measure includes the site and substance selection, the development of a multi-residue method to quantify pesticide residues in soil, the derivation of soil protection values, and the development of bioindicators for the effects of pesticides on soil fertility.

Soil represents one of the most complex environmental matrices and interactions of pesticides with it take place via divers binding processes [20, 21]. A fraction of applied pesticides can be irreversibly bound to the mineral and/or organic matter fraction, the so-called non-extractable residues (NER). Whether, and to which extent, NER are permanently incorporated into the soil matrix or if changing environmental conditions can lead to a time-delayed re-mobilization is still under debate and difficult to test [22, 23]. Therefore, within the total pesticide pool, it is crucial to distinguish between NER, the total extractable concentration (TEC) that is analytically accessible, and the bioavailable concentration, for which different concepts and definitions are in use [24]. The vast majority of studies, which focuses on the monitoring of pesticides in agricultural soils, relies on the TEC based on extensive extraction with organic solvents.

In contrast to other organic (e.g., polycyclic aromatic hydrocarbons and polychlorinated biphenyls) and inorganic (e.g., heavy metals) pollutants [25], and to the best of our knowledge, no aged certified reference material (CRM) exists, which would contain a large number of currently used pesticides. Therefore, during the validation of soil extraction methods, it is common practice to use soils that are spiked with the target analytes shortly before extraction. However, recoveries based on freshly spiked soil samples are expected to be clearly higher compared to those in aged soils. The reason for this is that interactions of analytes with the soil matrix largely differ between aged and freshly spiked soils due to the complex and increasingly strong binding processes of pesticides with the soil matrix as outlined above. Only aged soils can reflect extraction efficiencies comparable to those of field soils. Nevertheless, according to different guidelines on analytical method validation, the use of incurred materials, i.e., materials, in which the target analytes are initially alien but have been introduced before sampling (in the following called soils with native pesticides), is strongly recommended and the different extraction efficiencies from these and spiked materials are emphasized, but due to the lack of such CRM, its use is not mandatory [26, 27].

The introduction of QuEChERS [28] in the year 2003, an extraction method that stands for quick, easy, cheap, effective, rugged, and safe, originally developed to extract pesticides from fruits and vegetables, has largely superseded the more conventional time-consuming and/or high solvent volume-demanding methods to extract pesticides from soil, such as Soxhlet extraction, accelerated solvent extraction (ASE), ultrasonic-assisted extraction, or microwave-assisted extraction [29, 30]. Today, there are two different commonly used buffered QuEChERS methods, the European Committee for Standardization (CEN) Method EN 15662 using citrate buffer [31] and the AOAC Official Method 2007.01 using acetate buffer [32].

QuEChERS in combination with liquid (LC) or gas chromatography (GC) coupled to tandem mass spectrometry (MS/MS) has proven to be efficient and simple with satisfying analytical performance, as several recent multi-residue studies dealing with the quantification of pesticides in soil have demonstrated [8–11, 13, 15, 16, 18]. However, multiple analytical aspects were not or only partly addressed in the before mentioned studies. These include (i) method validation, which was in all studies only based on soils spiked with a known amount of analytes shortly before extraction and did not consider aged soils, i.e., soils that contained native pesticide residues, (ii) partly lacking sensitivities preventing the assessment of long-term ecotoxicological effects in the sub-ng/g range (method limits of quantification (MLOQ) 1–20 ng/g [8–10, 15, 16]), and (iii) quantification relying on (a) a limited number of isotopically labeled internal standards (ILIS) [9, 10, 18], (b) no ILIS at all [8, 13, 15], or (c) ILIS that were only added into the final extract not compensating analyte losses during the extraction process [10]. When using LC-MS/MS with electrospray ionization (ESI), quantifying without ILIS is acceptable if quantification is based on matrix-matched calibration using soil with similar soil characteristics (and thus similar matrix effects during ESI) compared to the analyzed field soil samples. However, in routine soil monitoring with the need of analyzing many different soils and sites, ILIS are essential for quantification regardless of the soil type. Finally, (iv) only two studies offer the quantification of more than 100 pesticide residues in soil [13, 16], whereas the remaining studies comprise between ~ 30 and 80. Regarding the huge number of different synthetic-organic pesticide active ingredients approved as plant protection products in the European Union and in Switzerland (between 200 and 250 [33, 34]) and the occurrence of pesticides in agricultural soils much longer than predicted by their half-lives [35], a multi-residue approach covering as many pesticides as possible should be sought. In this way, the risk associated with pesticides can be evaluated and characterized most comprehensively.

Within this study, a multi-residue QuEChERS-based LC-ESI-MS/MS (triple quadrupole) method to quantify 146 pesticides (60 fungicides, 30 herbicides, 26 insecticides, 4 acaricides, 4 rodenticides, 2 plant growth regulators, 1 synergist, and 19 transformation products (TPs); the status of approval as plant protection product in Switzerland according to the *Ordinance on Plant Protection Products, SR-916.161* [34] is listed in ESM-A Table S2) in soil was developed. Emphasis was put on (i) method validation in light of lacking aged CRM with native pesticide residues, (ii) sensitivity, to quantify pesticide residues in the sub-ng/g range, (iii) quantification confidence and the use of ~ 100 ILIS to compensate potential analyte losses during sample preparation (extraction and further sample treatment) and especially soil-specific matrix effects during ESI in view of the need to analyze many different soils with varying soil properties, and (iv) a multi-residue approach, i.e., the quantification of a large number of pesticides that are of relevance for a long-term soil monitoring. Its applicability is exemplified with analyses of a selection of different Swiss (agricultural) soils.

## Materials and methods

### Chemicals and solutions

Detailed information (CAS Registry Number, vendor, and purity) about all 146 analytes and 95 ILIS are provided in ESM-A Table S1.1. Other chemicals and solvents are listed in ESM-A Table S1.2.

An in-house made analyte MIX solution of exact concentration (250 ng/mL) was prepared gravimetrically in acetonitrile. In addition, two external analyte MIX solutions (ordered at *LGC Standards Ltd*. (Teddington, UK) and from the *Laboratory of the Canton of Zurich* [36]) were used for quality control. Details concerning the different analyte MIX solutions are given in ESM-B 1.

Similarly, an in-house made ILIS MIX solution of exact concentration was prepared in acetonitrile. The concentration of each ILIS in the MIX solution was adjusted based on analytical sensitivity; ILIS were assigned to three concentration levels resulting in one ILIS MIX solution with concentrations of 50–250–750 ng/mL. In this way, amounts of highly expensive ILIS were reduced, as spike levels need to be adapted to the least sensitive of them.

### Pesticide selection

In general, all active ingredients (organic and inorganic) and thereof major TPs (in case information to the below-mentioned criteria was available) were considered that were approved as plant protection products in Switzerland mainly between 2012 and 2019 [34] (~ 2700 candidates). Pesticides relevant for a long-term soil monitoring (i.e., pesticides likely to remain as residues) were then selected based on three categories: (i) their application (frequency and amount of usage in Switzerland), (ii) their environmental behavior (soil degradation measured in terms of half-lives (DT_50_) and mobility measured in terms of organic carbon-water partition coefficients (K_OC_)), and (iii) their ecotoxicity (toxicity measured in terms of acute or chronic studies and bioaccumulation potential measured in terms of octanol-water partition coefficients (log K_OW_)). Points were assigned per category (i–iii) if specific criteria were fulfilled and the detailed selection procedure is presented in ESM-B 2.

Based on this approach, 145 pesticides were classified as relevant and potentially LC-ESI-MS/MS multi-residue capable, and 120 of them were included in the final analytical method. The main reasons why 25 pesticides were excluded are: (i) their hydrophobicity (logK_OW_ up to 7), meaning that ionization by ESI is hindered, (ii) fast hydrolysis, (iii) no selective ion transitions for a MS detection (triple quadrupole), and (iv) unavailability of reference standards for method development. Additionally, all pesticides of the former analytical method of our laboratory [6] were added to the final analytical method to maintain comparability of past and future pesticide measurements, although some of these pesticides (*n* = 23) did not pass the selection procedure. Finally, three more pesticides were added due to expert opinions. Thus, in total 146 pesticides, including 127 parent substances and 19 TPs, are contained in the final analytical method (see ESM-A Table S2).

### Soil samples

Table 1 summarizes the used soil samples during method development (i.e., for optimization and validation) and application along with their main characteristics. Standard soils received from LUFA (*Landwirtschaftliche Untersuchungs- und Forschungsanstalt Speyer*, Deutschland) (S1 and S2) and from WEPAL, Wageningen University (*Wageningen Evaluation Programs for Analytical Laboratories*, the Netherlands) (S3 and S4) were used during method development. Soil S2, which only contains traces (< MLOQ) of six target pesticides and which is largely representative of Swiss agricultural soils, was used for matrix-matched calibration (see “Quantification”).

**Table 1:** Soils used for method development and application

Soil	Soil type	Land use	Organic carbon (C_org_) content [%]^c^	pH (0.01 M CaCl_2_)
S1	Loamy sand, standard soil LUFA^a^ 5M	-	0.89	7.4
S2	Clayey loam, standard soil LUFA^a^ 2.4	-	2.0	7.4
S2.1	Clayey loam, standard soil LUFA^a^ 2.4, spiked with all target analytes and partly aged (see “Soil samples”)	-	2.0	7.4
S3	Forest sandy soil, WEPAL^b^ ISE 867	-	5.1	3.6
S4	Loamy soil, WEPAL^b^ ISE 865	-	3.9	4.4
S5	Loamy clay	Crop land	2.8	6.8
S6	Silt	Crop land	1.3	6.5
S7	Loam	Crop land	1.4	6.1
S8	Sandy loam	Crop land	1.1	6.4
S9	Clay	Crop land	14	6.1
S10	Silt	Crop land	1.2	7.3
S11	Clayey sand	Vegetable site	1.5	7.4
S12	Loam	Orchard	2.3	5.5
S13	Loam	Vineyard	1.4	6.9
S14	Loamy sand	Vineyard	4.0	5.9
S15	Clayey loam	Grassland site	4.3	6.2
S16	Loam	Grassland site	3.9	5.6
S17	Sandy loam	Municipal park	2.0	6.7
S18	Sandy loam	Swiss national park	4.0	5.0

^a^LUFA: Landwirtschaftliche Untersuchungs- und Forschungsanstalt Speyer, Deutschland

^b^WEPAL: Wageningen Evaluation Programs for Analytical Laboratories, the Netherlands

^c^C_org_ contents of S5 to S18 were determined according to the Swiss reference methods of the research institute Agroscope [37] with the modified Walkley-Black method. C_org_ contents of S1 to S4 were determined by the suppliers and were used as received

Soil S2 was also used to prepare a reference soil, which contains all target analytes (S2.1). One kilogram of S2 was suspended in a volume corresponding to 300% water holding capacity (WHC) (45.8 ± 2.7 g/100 g) using 10 mM CaCl_2_ solution and was spiked with all target analytes to a concentration of 10 ng/g dry weight. This WHC created a free-flowing soil-water suspension, allowing for turbulation and efficient equilibration of the spiked analytes, which was then mixed for 7 days in the dark using a *TURBULA ®* shaker mixer (*TURBULA*; *Willy A. Bachofen AG,* Muttenz, Switzerland). The suspension was not sterilized to maintain the soil structure and the soil characteristics. Instead, mixing at 5 ± 1 °C was chosen to minimize microbial degradation of the analytes. In the next step, the soil was freeze-dried, sieved again (≤ 2 mm), and *turbulated*. Finally, the prepared soil was stored in an amber glass bottle at − 20 °C in the dark.

The prepared reference soil S2.1 is considered partly aged. Pesticides had more time to interact with the soil matrix compared to freshly spiked soils as suspending, mixing, and freeze-drying were supposed to contribute to soil aging; however, pesticides in field soil samples can outlast for decades depending on their soil dissipation and certainly undergo stronger binding processes compared to those in S2.1. Nevertheless, in this way, method validation of all target analytes in terms of trueness and inter-day method precisions was not only based on relative recoveries of freshly spiked soil samples, but was complemented by the recoveries of the partly aged reference soil S2.1.

Additionally, soil samples from Swiss agricultural sites with native pesticide residues were used during method development (S5 to S10) and application (S5 to S8 and S11 to S16). In addition, soils from two sites without agricultural usage, i.e., a Swiss municipal park (S17) and the Swiss national park (S18), were included as “negative controls". Representative soil sampling (all top soils, 0–20 cm) and subsequent pre-treatment as well as storing conditions are described in ESM-B 3.

### Soil extraction

QuEChERS was selected to extract pesticides from soil samples. The used method is based on the QuEChERS AOAC method [32] including some modifications (sample amount, volume and type of extraction agent while maintaining the sample amount to solvent volume ratio (1 g:1 mL), additional sonication and mixing steps, and the omission of any sample clean-up). Before starting the extraction, the distributional heterogeneity of dried and sieved soil samples was minimized using a *TURBULA*. Then, aliquots of 5 g ± 0.02 g soil were weighed into 50 mL plastic centrifuge tubes. In the next step, 100 µL ILIS MIX solution (50–250–750 ng/mL) was spiked directly onto each weighed-in soil sample, and the organic solvent of the spiked ILIS MIX solution was allowed to evaporate for at least 1 h. Then, 5 mL *nanopure*-H_2_O was added to each soil sample, after which they were shortly vortexed (~ 10 s) and then mixed for 15 min using a *TURBULA*. Water was added to the dried soil samples to aid in extracting by making the pores more accessible to the extraction solvent. Subsequently, 5 mL of acidified acetonitrile (2.5% formic acid (FA)) was added and the samples were shortly vortexed (~ 10 s). Mixing of *nanopure*-H_2_O and acetonitrile is an endothermic reaction; to release the pressure present in the plastic centrifuge falcon tube and to avoid a potential leaking during mixing, the centrifuge tubes were once shortly opened and closed again. Soil samples were then mixed for 15 min using a *TURBULA*. In the next step, samples were sonicated for 10 min, then shortly vortexed (~ 10 s) and sonicated again for 10 min (720 W). Following that, a salt mixture composed of 4 g magnesium sulfate and 1 g sodium acetate was added and the samples were immediately vortexed for 1 min, mixed using a *TURBULA* for 15 min, and sonicated for 10 min. Samples were then centrifuged (Rotanta 460R, *Hettich GmbH & Co. KG*, Tuttlingen Germany) for 4 min at 4000 rpm (1788 rcf). Finally, 1 mL of each supernatant was transferred unfiltered to LC vials for chemical analysis.

Soils S5 to S8 were additionally analyzed undried (see ESM-B 3) in quadruplicate. Therefore, they were mixed using a *TURBULA* before taking aliquots and the processed amount of undried soil (5 g ± 0.02 g) was adjusted according to the determined water content (between 16 and 25%) to achieve a similar initial weight based on dry weight.

Method performance criteria such as extraction efficiencies and intra-day method precisions of the developed QuEChERS method were compared with those of a previously applied and slightly adapted accelerated solvent extraction (ASE) method developed in our laboratory [6] (for details refer to ESM-B 4).

### Chemical analysis using LC-ESI-MS/MS

Samples were measured on a LC-ESI-MS/MS instrument. Sample injection into a 5 µL sample loop (4 times overfilling) was carried out using a PAL RTC autosampler (*CTC Analytics AG*, Zwingen, Switzerland). For chromatographic separation, a Kinetex Biphenyl 100 Å column (100 × 4.6 mm, 5 µm particle size, *Phenomenex,* Torrance, USA) equipped with a C_18_ pre-column (4 × 2 mm, *Phenomenex,* Torrance, USA) was used. The mobile phase consisted of *nanopure*-H_2_O and methanol, both containing 5 mM NH_4_COOH, and LC was performed at 35 °C at a flow rate of 750 µL/min (Infinity 1290, *Agilent Technologies*, Palo Alto, USA). The LC run lasted 29 min and the optimized mobile phase gradient is presented in ESM-A Table S3.1. Ionization was carried out by ESI in positive and negative mode and detection was performed with a triple quadrupole MS (QTrap 5500, *Sciex*, Toronto, Canada) using the scheduled multiple reaction monitoring scan mode. A mass resolution of 0.7 ± 0.1 Da was selected for Q1 and Q3 (unit isolation mode). The target cycle time was set to 0.6 s resulting in dwell times from 4.6 ms to 167 ms for each ion transition (median dwell time of 13 ms).

For each analyte and ILIS, two ion transitions were acquired leading to in total 484 ion transitions, of which the more sensitive ion transition per analyte and ILIS was used as quantifier and the less sensitive one as qualifier ion transition. All relevant MS/MS parameters are listed in ESM-A Tables S3.2 to S3.5.

### Quantification

Soil S2 (standard soil LUFA 2.4, see Table 1 for soil characteristics) was used to prepare matrix-matched calibration curves. Simultaneously to each batch of (field) soil samples, twice 10 g of S2 was extracted as described in “Soil extraction” with the exception that no ILIS MIX solution was added before the extraction and that the double amount of solvents (*nanopure*-H_2_O, acetonitrile (2.5% FA)) and salts was used. The soil extracts of both S2 samples were combined and mixed. Matrix-matched calibrations standards with concentrations of 0.05–0.1–0.25–0.5–1–2.5–5–10–17.5–25–35–50 ng/mL (equivalent to ng/g) were prepared (final ILIS concentration in each calibration standard 1, 5 or 15 ng/mL; details are given in ESM-B 5).

Quantification (concentrations expressed as ng/g dry weight) was based on matrix-matched *internal standard calibration* (MultiQuant Quantitative Analysis software 3.0.3, *Sciex*, Toronto, Canada) using linear least square regression with a weighing factor of 1/x. To construct calibration curves, the peak area ratio (PAR: peak area of the analyte divided by the peak area of the corresponding ILIS) of each substance in each calibration standard was plotted against the corresponding concentration level. If stereoisomers were chromatographically separated (see ESM-A Tables S3.3 and S3.4), the peak areas of all stereoisomers were integrated and summed. Structure-identical ILIS (si-ILIS) were available for 95 of the 146 analytes. For analytes with si-ILIS (analyte_si-ILIS_), solvent-based instead of matrix-matched internal standard calibration would lead to equally accurate quantification. However, MLOQ determination is hindered (see “Method validation”) and potential signal interferences of analytes by matrix overlays are overlooked. For the remaining analytes (analyte_nsi-ILIS_), non-structure-identical ILIS (nsi-ILIS) were used for quantification and ILIS were selected that ideally led to relative recoveries between 70 and 120% [27] in all tested soils (S1 to S5). To this end, the raw data from the relative recovery experiment (see “Method validation” and ESM-B 9) was used, in which the soils S1 to S5 were analyzed spiked (2.5 ng/g) and unspiked. The systematic procedure to select ILIS for each analyte without si-ILIS (analyte_nsi-ILIS_) is described in detail in ESM-B 6.

Average qualifier-to-quantifier ion ratios (peak area of the qualifier ion transition divided by the peak area of the quantifier ion transition) were calculated for each analyte in all matrix-matched calibration standards in every sequence. Then, the qualifier-to-quantifier ion ratio of each analyte in the simultaneously analyzed soil samples was compared to the average qualifier-to-quantifier ion ratio. Qualifier-to-quantifier ion ratios of each analyte in the soil samples were allowed to deviate from the corresponding average qualifier-to-quantifier ion ratio according to the criteria defined in the EC Directive 2002/657/EC [38] and listed in ESM-B 7.

### Method validation

To the best of our knowledge, no guidance document exists, which specifically deals with the method validation of pesticide analysis in soil. Thus, the *SANTE 11312/2021* guideline on *Analytical quality control and method validation procedures for pesticide residue analysis in food and feed* [27] was followed. Definitions of validation-related terms can largely vary across different guidelines, especially concerning the terms accuracy, trueness, and precision. Within this study, accuracy is defined as the combination of trueness and precision as specified by ISO 5725-4:2020 [39].

The optimized QuEChERS method was validated in terms of absolute recoveries covering the QuEChERS extraction (absolute recoveries_QuEChERS_), trueness, different precisions, linearity, selectivity, matrix effects, MLOQs, and instrumental limits of quantification (ILOQs).

Absolute recoveries_QuEChERS_ were determined based on S1 to S3 (spike level: 2.5 ng/g) to identify analyte losses during the extraction process, which influence the performance of the developed method, e.g., in terms of sensitivity (for details refer to ESM-B 8).

Trueness was assessed by the analysis of external reference standards (see “Chemicals and solutions”), the repeated analysis of S2.1 (16 sample preparations in duplicate within 6 months), and relative recoveries of freshly spiked soils (S1 to S5 using different spike levels between 0.1 and 10 ng/g, for details refer to ESM-B 9), as well as by participating in a ring trial (*PT-PAS-II: Determination of Pesticides in Agricultural Soil*, organized by the Central Institute for Supervising and Testing in Agriculture (ÚKZÚZ), Department of Proficiency Testing Programmes (OdMPZ), in April 2022).

Different precisions, i.e., instrumental precisions, intra-day method precisions (repeatability), inter-day method precisions (intermediate precision), and inter-person method precision were determined as detailed in ESM-B 10.

Linearity of the matrix-matched calibration curves was reviewed by comparing the calculated concentration with the spiked/actual concentration of each calibration standard (allowed deviation  ≤  ± 20%). Selectivity was ensured by acquiring two characteristic ion transitions for each analyte and ILIS (for details, see ESM-B 7), by reviewing the qualifier-to-quantifier ion ratio (see “Quantification”), and by matching retention times (± 0.05 min) of each analyte and ILIS in the field soil sample with those in the simultaneously acquired matrix-matched calibration standard.

To determine soil-specific matrix effects, S1 to S5 were extracted as described in “Soil extraction” with the exception that ILIS and analyte MIX solution (final concentration in the LC-vial: 2.5 ng/mL) were added to the soil extracts after the extraction. The soil extracts of S1 to S5 were each analyzed twice spiked with analyte and ILIS MIX solution and each twice only spiked with ILIS MIX solution. Additionally, two calibration standards with concentrations of 2.5 ng/mL were prepared in acetonitrile (2.5% FA). Matrix effects of S1 to S5 were calculated as follows, either using the average signal intensities in the calibration standards prepared in acetonitrile (2.5% FA) as reference *(case (i))* or using the average signal intensities in the S2 extracts as reference *(case (ii)):*

1 $$Matrix\,effect\left[\%\right]=\left(1-\frac{{\overline{PA}}_{spiked\;Sx\;extract}-{\overline{PA}}_{unspiked\;Sx\;extract}}{reference\,(case\,\left(i\right)\,or\;case\,\left(ii\right))}\right)\ast(-100)$$

where $\overline{PA}$ is the average peak area of each analyte, reference *case (i)* stands for ${\overline{{P}{A}}}_{{c}{a}{{l}}{i}{b}{r}{a}{t}{i}{o}{n} \,{s}{t}{a}{n}{d}{a}{r}{d}\, {p}{r}{e}{p}{a}{r}{e}{d} \,{i}{n}\, {a}{c}{e}{t}{o}{n}{i}{t}{r}{i}{l}{e}\, (2.5\mathrm{\% }\,{F}{A})}$, and reference *case (ii)* stands for ${\overline{{P}{A}}}_{{s}{p}{i}{k}{e}{d}\, {S}2 \,{e}{x}{t}{r}{a}{c}{t}}-{\overline{{P}{A}}}_{{u}{n}{s}{p}{i}{k}{e}{d}\, {S}2 \,{e}{x}{t}{r}{a}{c}{t}}$.

In this way, matrix effects are expressed as positive and negative percentages, at which positive values indicate ion enhancement and negative values ion suppression during ESI.

Substance-specific MLOQs were defined as the concentration of the matrix-matched calibration standard, for which the analyte peak area of the quantifier and qualifier ion yielded signal-to-noise (S/N) ratios of at least 10 and 3, respectively, and for which the qualifier-to-quantifier ion ratio matched the expected one. They were based on a soil amount of 5 g and an extraction volume of 5 mL. In the next step, MLOQs were adjusted by a *global matrix correction factor*, which was deducted based on the matrix effects generated by the soil extracts of S1 to S5 (organic carbon (C_org_) contents between 1 and 5%, see Table 1). The reason why to assign this *global matrix correction factor* is described in detail in “Limits of quantification.” Blank contamination was considered by analyzing almost target-pesticide-free S2 samples (see “Soil samples”) simultaneously to the analysis of field soil samples. Target pesticides were never detected in any S2 blank sample above MLOQ.

Instrumental LOQs were determined by injecting analyte MIX standards of concentrations 0.005, 0.01, 0.025, 0.05, 0.1, 0.25, 0.5, 1, and 2.5 ng/mL in pure solvent (acetonitrile acidified with 2.5% FA) and the chromatographic peaks had to fulfil the same S/N criteria as mentioned above.

### Data visualization and evaluation

All figures were created with R version 4.2.2. [40]. To state significant differences between two groups (e.g., individual pesticide concentrations either obtained by QuEChERS or by ASE), their average values were compared with a *t*-test (two-tailed distribution, two-sample equal variance (homoscedastic), *p* < 0.05). The equal variance between the two groups was tested beforehand with an F-test (two-tailed probability that the variances in group 1 and group 2 are not significantly different). Percentage differences between different groups were calculated as follows:

2 $$Difference\, \left[\%\right]= \frac{result\, by\, group\, x - result\, by\, group\, y}{result\, by\, group\, x}*100$$

For the comparison of pesticide concentrations either obtained by QuEChERS or by ASE, a linear model was fitted in addition to the applied *t*-tests.

## Results and discussion

### Method optimization

#### LC-MS/MS

The optimization and review for selectivity of all ion transitions, collision energies and potentials as well as the optimization of the interplay of LC (peak width), detection window, target cycle time, and dwell times are described in detail in ESM-B 11. Information concerning the in total 484 ion transitions included in the final MS/MS acquisition method as well as ESI source and gas parameters are listed in ESM-A Tables S3.2 to S3.5.

#### Selection of a suitable extraction method

In comparison to the QuEChERS AOAC method [32], the sample amount to solvent volume ratio (1 g:1 mL) was maintained but the original sample quantity of 15 g was reduced to 5 g. This amount of soil proved sufficient in terms of sample representativeness, sensitivity, linear range (see “Method validation”), and the necessity to add ILIS before the extraction. The advantage of adding ILIS before the extraction is reflected by on median 7% higher relative recoveries than absolute recoveries_QuEChERS_ (significant difference for 69% of in total 428 detects in the spiked soils S1 to S3, individual differences are listed in ESM-A Table S4.1; the method validation parameters relative recovery and absolute recovery_QuEChERS_ are presented in “Absolute recoveries_QuEChERS_” and “Trueness”) and increased pesticide concentrations in S8 to S10 and S2.1, when ILIS was added before the extraction. Especially atrazine-2-hydroxy, metolachlor ESA, metolachlor OA, and thiabendazole were susceptible to considerable losses during the extraction and further sample preparation in all tested soils and adding ILIS before the extraction increased the quantified pesticide concentrations by up to 80% (individual differences are listed in ESM-A Table S4.1).

Acidic conditions were identified to increase the extraction efficiencies of pesticides from the soil during ASE [7], which is why the QuECHERS AOAC [32] method was given preference over the one from CEN [31]. According to Acosta-Dacal et al. [13], the extraction efficiencies were additionally increased when using the extraction agent acetonitrile (2.5% FA, pH ~ 3.8) instead of using the original AOAC QuEChERS extraction agent of acetonitrile (1% acetic acid, pH ~ 4.2). This finding was confirmed within this study. A median increase in individual pesticide concentrations of 13% (significant difference for 86% of in total 142 detects, individual differences are listed in ESM-A Table S4.2) was achieved for S2.1 when extracted with acetonitrile (2.5% FA) instead of acetonitrile (1% acetic acid). Correspondingly, quantified individual pesticide concentrations in S8 to S10 were on median by 19% higher (significant difference for 55% of in total 99 detects, individual differences are listed in ESM-A Table S4.2).

The extraction of pesticides from the soil was assisted by additional sonication and mixing steps [11, 13]. Absolute recoveries_QuEChERS_ as a measure of extraction efficiencies were satisfying and are presented in “Absolute recoveries_QuEChERS__”._

The original QuEChERS AOAC method utilizes a sample clean-up (typically dispersive solid-phase extraction using magnesium sulfate and primary secondary amine sorbent). Co-extracted matrix constituents certainly affected the signal intensities of the target analytes when using LC-ESI-MS/MS (see “Matrx effects during ESI”). However, it had been shown that sample clean-up can also lead to losses in recovery, especially for more polar pesticides [13, 41]. Additionally, due to excellent instrumental sensitivities when working with an injection volume of 5 µL (median matrix-matched ILOQ using S2 soil matrix: 0.1 ng/mL), no clean-up of the soil extracts was necessary keeping the extraction method as simple as possible.

In the next step, extraction efficiencies (measured in terms of obtained concentrations) and intra-day method precisions of the optimized QuEChERS extraction method were compared with those of the ASE method [6] (for details refer to ESM-B 4) previously applied in our laboratory to quantify pesticides in soil for S2.1 and S8 to S10. Matrix-matched calibration was employed for both methods. For ASE, due to a large extraction end volume, ILIS had to be added after the extraction thereby not compensating potential analyte losses during the extraction and further sample preparation. However, absolute recoveries_ASE_ covering the extraction process were between 70 and 120% for 93% of all analytes (individual data not shown) pointing towards little analyte losses, thereby allowing for a comparison of obtained concentrations, although ILIS were added before (QuEChERS) and after (ASE) the extraction, respectively.

**Fig. 1:**
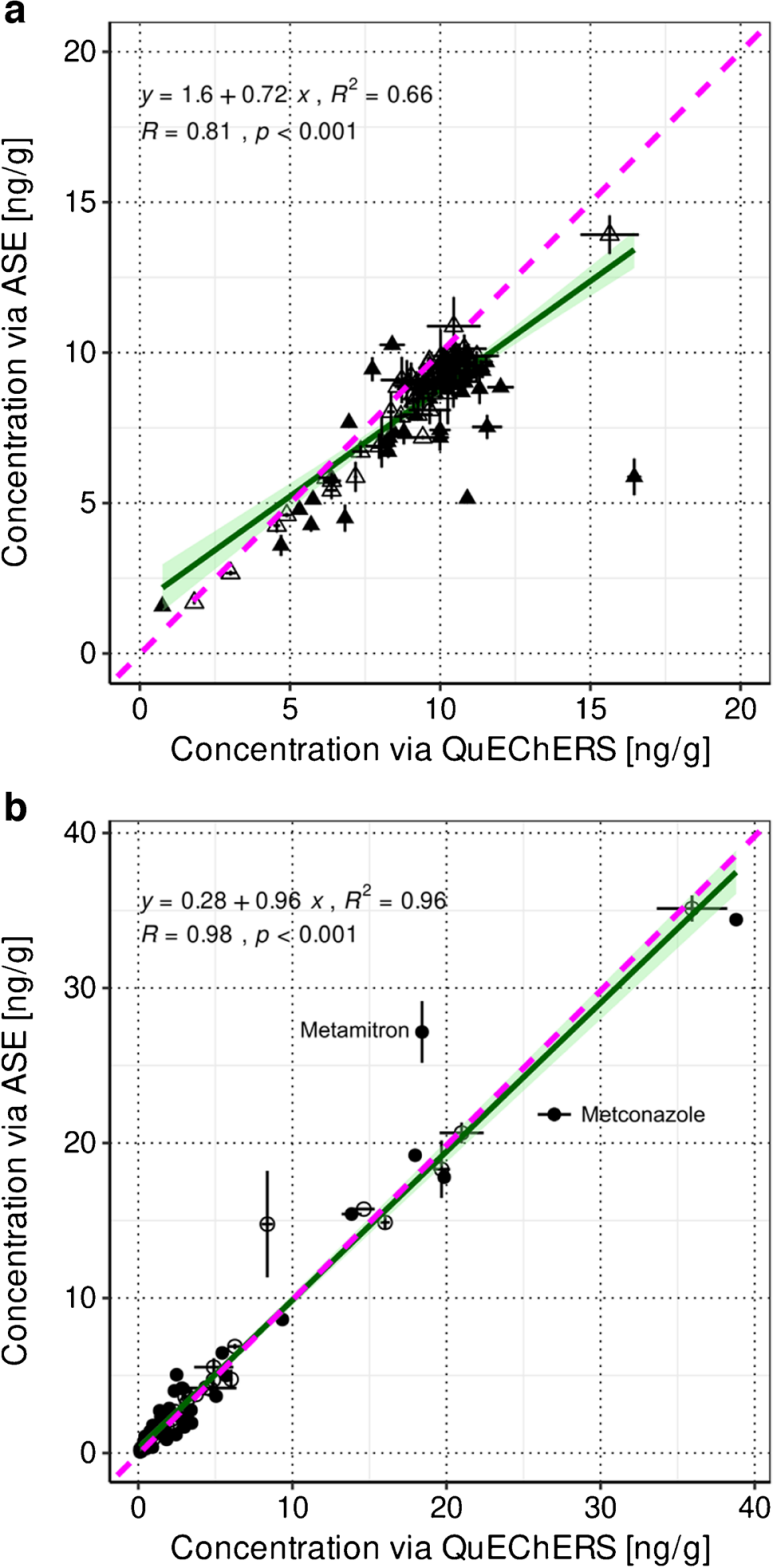
1:1-line plot of the average individual pesticide concentrations quantified in soil S2.1 (142 detects, panel **a**) and in soils S8 to S10 (103 detects, panel **b**), extracted with QuEChERS and with ASE (number of replicates for both extraction methods *n* = 2 except for S8 and S9 extracted with ASE *n* = 3). In case the difference of the individual pesticide concentration based on a *t*-test (two-tailed distribution, two-sample equal variance (homoscedastic), *p* < 0.05) between the ASE and the optimized QuEChERS method was not significant, corresponding detects are displayed with open symbols. The pink dashed lines depict the lines of identity and the green lines represent the linear regression lines with their 95% confidence interval. Additionally, the linear equations together with their *R*^2^ and the Pearson correlations (*R* and *p*-value) are displayed

With QuEChERS, a median increase in individual pesticide concentrations of 10% was achieved in S2.1 compared to the concentrations obtained by ASE (significant difference for 49% of in total 142 detects, individual differences are listed in ESM-A Table S4.3), which is also reflected in the slope of the linear regression line (0.72) (see Fig. 1a). For S8 to S10, the slope of the linear regression line is close to one (see Fig. 1b) pointing towards comparable extraction efficiencies between QuEChERS and ASE also in agricultural soils with native pesticide residues (individual differences are listed in ESM-A Table S4.3). These findings are in line with previous studies [11, 42, 43] dealing with the assessment of extraction efficiencies by QuEChERS and ASE. Moreover, intra-day method precisions did not differ for both extraction methods and were on median 2% and 4% for S2.1 and S8 to S10, respectively (for intra-day method precisions of the QuEChERS method, also see “Precision”).

However, when working with multi-residue methods, there are certainly individual substances that show better extraction efficiencies with either of the methods, e.g., metamitron (ASE) or metconazole (QuEChERS) (see Fig. 1b). Multi-residue methods are always a compromise and an appropriate extraction method has to be chosen based on the results of the majority of all analytes.

#### Soil sample treatment before extraction

For convenience in terms of infrastructure and subsample availability, soil samples are preferentially stored under ambient or slightly cooled conditions in the dark. This however requires sample stabilization by drying beforehand. To investigate whether drying of soil samples at moderately elevated temperatures (40 °C) until constant weight may lead to pesticide losses, S5 to S8 were analyzed dried and undried, i.e., soil samples were directly frozen after sampling, slightly defrosted before sieving < 5 mm using liquid nitrogen, and stored frozen until analysis (for details, see ESM-B 3).

Dried and undried S5 to S8 showed similar results in terms of number (between 29 and 37 pesticides were detected in S5 to S8, no matter if analyzed dried or undried) and identity of the detected pesticides and in terms of the detected concentration ranges per substance (individual differences and concentrations for both treatments are listed in ESM-A Tables S4.4 and S5, respectively). The sum concentrations determined in soils treated in either way did not differ and were within intra-day method precision (median relative standard deviation (RSD) of the quadruplicate sample preparations per treatment (dried or undried soil samples) of all detected individual pesticide concentrations in the four dried/undried soil samples S5 to S8: 4%/7%; see also “Precision” for intra-day method precision based on dried soil samples, individual intra-day method precisions for both treatments are listed in ESM-A Table S11.2). In addition to the decrease in intra-day method precision when analyzing undried soil samples, intra-day method precision was on maximum 158%, and 21 of the in total 129 detects over all soils exhibited an intra-day method precision higher than 20%. In contrast, it was on maximum 38% for the dried soil samples and it was only higher than 20% for six of the detects. This indicates that dried soil samples are less heterogeneous than undried soil samples, which allows operating with smaller sample aliquots for analysis. Obviously, grinding and sieving < 2 mm in contrast to sieving < 5 mm decreases the soil heterogeneity. However, due to practical reasons, frozen and subsequently slightly defrosted samples are difficult to handle, and sieving through a smaller mesh size is technically not feasible.

Overall, this comparison justifies the extraction of dried soil samples without facing the risk of losing pesticides due to gentle drying and points towards similar extraction efficiencies of dried and undried soil samples. The main advantages of analyzing dried soils compared to undried soils are better intra-day method precisions, its practicability for sample preparation and storage as well as improved availabilities of subsamples, e.g., for repeated analyses. While the long-term stability of pesticides in stored samples largely remains to be tested, it has been proven to be the case at least for selected pesticides for up to eight years [35].

#### Matrix effects during ESI

**Fig. 2:**
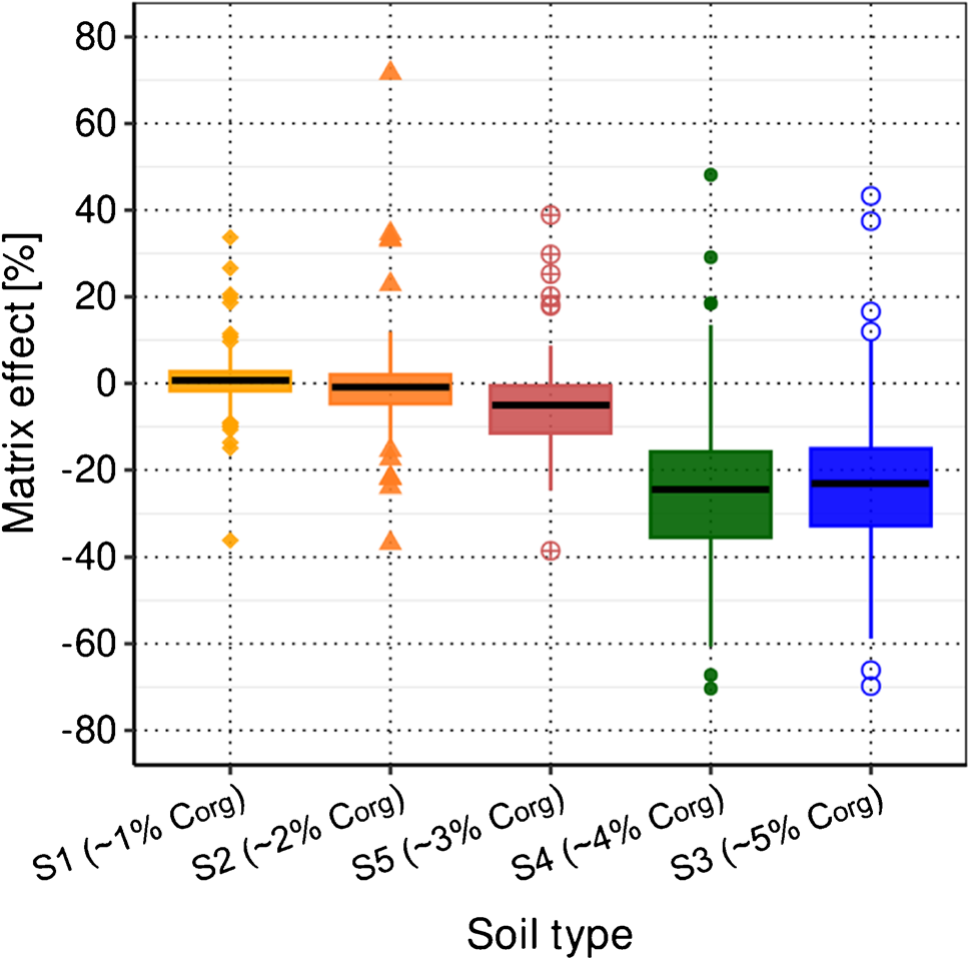
Boxplots of the matrix effects of all pesticides included in the developed analytical method in soils S1 to S5 with C_org_ contents between 1 and 5% using as reference the signal intensities of each analyte in a calibration standard prepared in acetonitrile (2.5% FA) (*case (i)*; for details, see “Method validation”)

Matrix constituents are co-extracted from the soil sample. Those that end up dissolved in the final extract, e.g., humic acids, transport through and co-elute with the analytes from the LC column, most likely altering the ionization efficiencies of the analytes during ESI, thus strongly influencing the sensitivity for each measured analyte. Therefore, matrix effects of S1 to S5 with C_org_ contents of ~ 1 to 5% were investigated to reflect the typical range of Swiss topsoil (0–20 cm) croplands [44]. Figure 2 shows the distribution of the matrix effects of all 146 pesticides in the soil extracts of S1 to S5 using the corresponding signal intensities in a calibration standard prepared in acetonitrile (2.5% FA) as reference (*case (i),* see “Method validation”). Median matrix effects ranged from 1% (S1) to − 24% (S4). These results confirmed the assumption that matrix effects (predominantly ion suppression) increased with increasing matrix complexity reflected by the C_org_ content. The phenomenon of ion suppression of analytes in the presence of complex matrix constituents during ESI is widely known and has been investigated in different sectors such as environmental [45, 46], pharmaceutical [47], bioanalytical [48, 49], and food sciences [50].

Additionally, matrix effects for S1 and S3–S5 were calculated using the signal intensities of each analyte in the S2 extract as reference (*case (ii)*; see ESM-B 12 Figure S5 and “Limits of quantification”). Median matrix effects did not differ strongly for *case (i)* and *case (ii)*. However, minimum and maximum matrix effects were decreased when using the signal intensities of each analyte in the S2 extract *(case (ii))* as a reference. Individual matrix effects for S1 to S5 using either the signal intensities in acetonitrile (2.5% FA) (*case (i)*) or those in the S2 extract (*case (ii)*) as reference are listed in ESM-A Table S7.

#### ILIS selection for analytes without structure-identical ILIS

si-ILIS were available for 66% of all target analytes. For analytes_nsi-ILIS_, nsi-ILIS were used for quantification. However, selecting the most suitable ILIS for analytes_nsi-ILIS_ is not straightforward and was carried out by a systematic evaluation of relative recoveries of all analytes_nsi-ILIS_ – ILIS combinations (within a certain retention time window, detailed criteria are described in ESM-B 6) in the spiked soils S1 to S5 (C_org_ contents between 1 and 5%, see Table 1), leading at best to relative recoveries between 70 and 120% [27] for each analytes_nsi-ILIS_ in every tested soil matrix.

The median number of possible analytes_nsi-ILIS_ – ILIS combinations for analytes_nsi-ILIS_ that ionized in ESI + (*n* = 40) using a retention time window of ± 2 min around the retention time of each analyte was 26 and ranged between 4 and 32. For analytes_nsi-ILIS_ that ionized in ESI- (*n* = 11), eight analytes_nsi-ILIS_ – ILIS combinations were possible per analyte since only eight ILIS that ionized in ESI- were included in the analytical method and no retention time restrictions were applied.

**Fig. 3:**
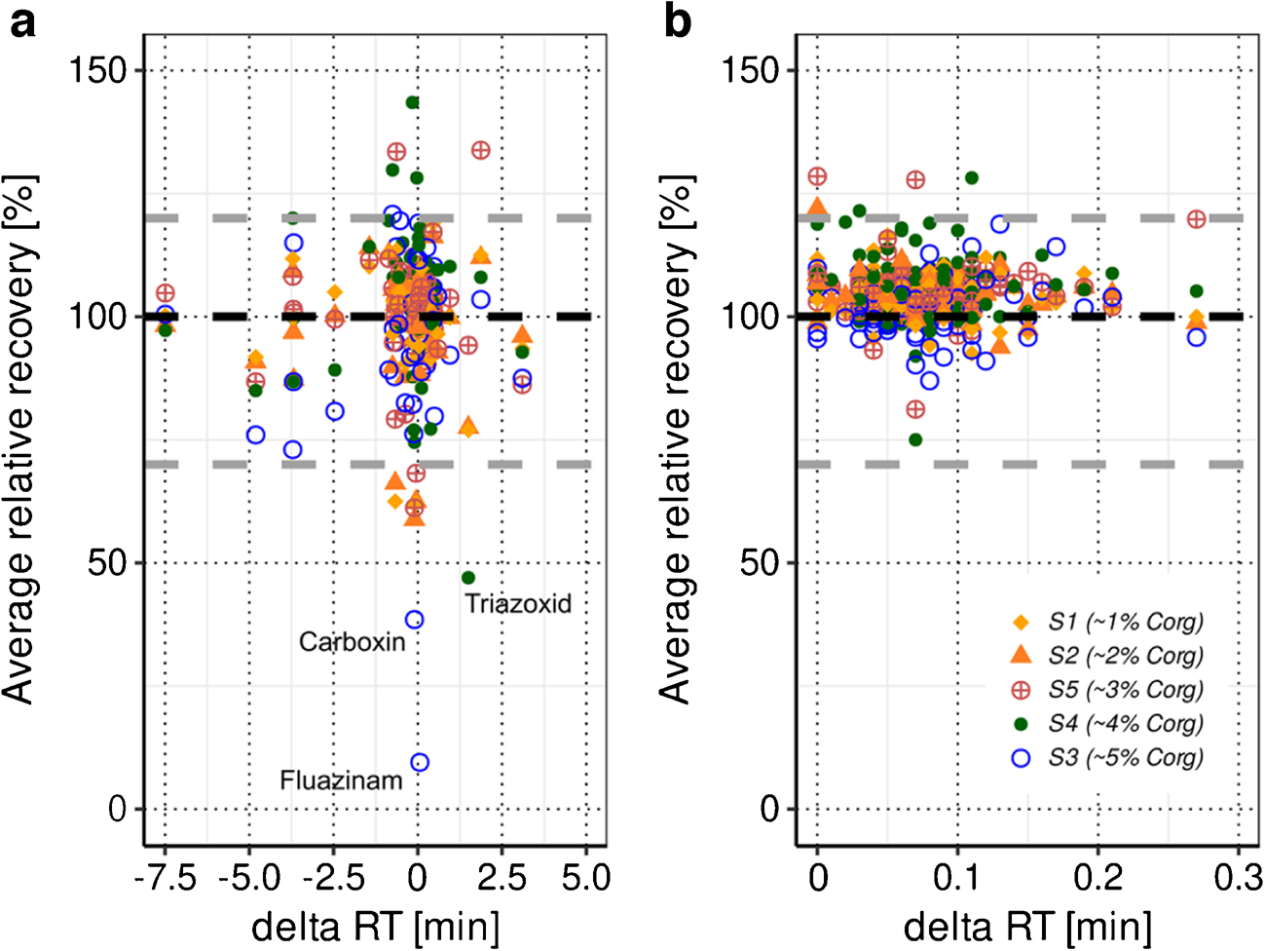
Relative recoveries of pesticides based on the final selection of analyte – isotopically labeled internal standard (ILIS) couples for analytes_nsi-ILIS_ (analytes without structure-identical ILIS, panel **a**, *n* = 51 analytes) and for analytes_si-ILIS_ (analytes with structure-identical ILIS, panel **b**, *n* = 95 analytes). Displayed are the average relative recoveries (quadruplicate sample preparations for soils S1 to S5) for soils S1 to S5 plotted against the retention time difference (delta RT [min]) of each analyte to the selected ILIS. The black dotted lines mark 100% relative recovery, whereas the grey dotted lines mark the requested range of relative recoveries (70–120%)

Figure 3 shows the relative recoveries based on the final selection of analyte – ILIS couples in S1 to S5 for analytes_nsi-ILIS_ (analytes without si-ILIS, panel A) and for analytes_si-ILIS_ (analytes with si-ILIS, panel B) for comparison. The depicted relative recoveries, which were used for the selection of ILIS for analytes_nsi-ILIS_, are based on one spike level (2.5 ng/g), and relative recoveries presented in “Trueness” refer to the three different employed spike levels (0.5, 2.5 and 10 ng/g, see ESM-B 9). All possible analytes_nsi-ILIS_ – ILIS combinations (ESI + and ESI-) based on the applied criteria are displayed in ESM-B 6 Figures S1 and S2.

For analytes_nsi-ILIS_, the median relative recovery over all soil types was 102%, and only 6% of all analyte – ILIS combinations led to average recoveries smaller than 70% or higher than 120%. For instance, the fungicide carboxin exhibited relative recoveries smaller than 70% for four out of the five tested soils. Carboxin is prone to fast soil degradation (0.5–3.3 days [51]) and none of the ILIS included in the analytical method could entirely compensate for this effect. Moreover, the fungicides triazoxide and fluazinam experienced strong ion suppression especially in the extracts of S3 and S4 with high C_org_ contents and no ILIS was able to compensate for this effect in all tested soil matrices (see Fig. 3a).

As expected, for analytes_si-ILIS_, 99% of the relative recoveries over all soils were within the requested range of 70–120% (median value 104%) and the median retention time difference between analytes_si-ILIS_ and ILIS was 0.07 min due to almost identical physico-chemical properties of ILIS compared to the unlabeled analyte. A comparable median retention time difference between analytes_nsi-ILIS_ and ILIS of − 0.07 min was observed for analytes_nsi-ILIS_, indicating that physico-chemical similarity (in terms of retention time) and therefore identical matrix exposure are key to effectively compensate ion suppression/enhancement. The final selection of ILIS for all analytes_nsi-ILIS_ is presented in ESM-A Table S6.

### Method validation

The final method optimized as described in “Method optimization” was thoroughly validated by means of absolute recoveries_QuEChERS_, trueness, precisions, linearity, and limits of quantification (ILOQs and MLOQs). Selectivity (addressed in “Method validation” and in ESM-B 7) and matrix effects (addressed in “Matrix effects during ESI” and “Limits of quantification”) indirectly affected these method validation parameters.

#### Absolute recoveries_QuEChERS_

Satisfying absolute recoveries_QuEChERS_ as a measure of extraction efficiencies were achieved with a median absolute recovery_QuEChERS_ of 95% considering S1 to S3 and only 4% of all determined absolute recoveries_QuEChERS_ were smaller than 70% (see Table 2). This points towards little analyte losses during the extraction and sample preparation (due to, e.g., sorption, thermal degradation, volatilization, or incomplete extraction efficiencies). Individual absolute recoveries_QuEChERS_ for all pesticides in S1 to S3 are displayed in ESM-A Table S8.

**Table 2:** Summarized figures of merit for the 146 pesticides included in the final multi-residue method for trace analysis of pesticides in soils

Figures of merit	Number of replicates	Summarized results	Individual results
**Extraction efficiency QuEChERS**			
Absolute recoveries_QuEChERS_^a^ based on S1 to S3	*n* = 4 for S1 to S3	Median: 95% > 70%: 96%	ESM-A Table S8
**Trueness**			
External reference standards	*n* = 1 for each analyte MIX solution	Median deviation of the three different analyte MIX solutions: 3%	Individual data not shown
Partly aged reference soil concentration S2.1	*n* = 16 (each in duplicate within 6 months)	Median: 9.3 ng/g deviation of more than 20% from spiked concentration of 10 ng/g: 27%	ESM-A Table S9
Relative recoveries^b^ based on S1 to S3	*n* = 4 for S1 to S3	Median: 103% between 70 and 120%: 97%	ESM-A Table S10
Ring trial	*n* = 2 for soil a and b	*z*-scores < 2 (median *z*-scores for soil a | b: 1.05 | 1.12)	Individual data not shown
**Precisions**			
Instrumental precision^c^	*n* = 5	Median: 2% (min: 0.5%, max: 10%)	ESM-A Table S11.1
Intra-day method precision based on spiked^b^ S1 to S3	*n* = 4 for S1 to S3	Median: 3% (min: 0.1%, max: 34%)	ESM-A Table S11.2
Intra-day method precision based on the agricultural field soils S5 to S8	*n* = 4 for S5 to S8	Median: 4% (min: 0.4%, max: 38%)	ESM-A Table S11.2
Inter-day method precision based on S2.1	*n* = 16 (each in duplicate within 6 months)	Median: 6% (min: 4%, max: 15%)	ESM-A Table S11.3
Inter-day method precision based on the agricultural field soils S5 to S8	*n* = 2 (each in quadruplicate within 3 months)	Median: 3% (min: 0%, max: 16%)	ESM-A Table S11.3
Inter-person method precision based on S2.1	Person 1 | 2: *n* = 9 | 7 independent sample preparations in duplicate within 6 | 4 months	Median: 2% (min: 0%, max: 11%)	ESM-A Table S11.4
**Linear range**		Median: 0.1 to 35 ng/g	ESM-A Table S6
**Matrix effects in S1 to S5**^d^	*n* = 2 for S1 to S5	Median: 1 to − 24% Maximum ion suppression: − 70 to − 39% Maximum ion enhancement: 34 to 74%	ESM-A Table S7
**Limits of quantification**			
ILOQ		Median: 0.025 ng/mL (min: 0.005 ng/mL, max: 2.5 ng/mL)	ESM-A Table S6
MLOQ		Median: 0.2 ng/g (min: 0.1 ng/g, max: 10 ng/g) ≤ 0.5 ng/g: 80%	ESM-A Table S6

^a^Spike level 2.5 ng/g

^b^Spike levels 0.5, 2.5, and 10 ng/g

^c^0.5 and 5 ng/mL matrix-matched calibration standards

^d^Using a calibration standard prepared in acetonitrile (2.5% formic acid) as a reference, *case (i)*, refer to “Method validation”

#### Trueness

In light of lacking CRM with native pesticide residues, trueness was determined at different levels of analytical complexity, i.e., (i) based on external reference standards (see “Chemicals and solutions”), (ii) the repeated analysis of the partly aged reference soil S2.1, (iii) relative recoveries of S1 to S3, and (iv) by participating in a ring trial.

The results based on S2.1 do not always directly represent trueness. The initially spiked concentration of 10 ng/g does not necessarily correspond to the target concentration for recovery since some pesticides are prone to NER formation or to fast soil degradation, such as the fungicide carboxin (DT_50_ ~ 0.5–3.3 days [51]), for which only ~ 0.7 ng/g was recovered. However, the median S2.1 concentration was 9.3 ng/g based on 16 independent duplicate analyses conducted over 6 months (see Table 2) and only 27% of the individual pesticide concentrations in S2.1 deviated more than ± 20% from the spiked concentration of 10 ng/g (individual S2.1 concentrations are listed in ESM-A Table S9). Moreover, quantified pesticide concentrations remained constant over 6 months (median inter-day method precision of 6%, see “Precision”). Thus, the averaged individual pesticide concentrations obtained from these repeated independent analyses of S2.1 can serve as target concentrations to review the accuracy (in terms of trueness and precision) of the method both for validation and for quality control under routine application for soil monitoring.

The median relative recovery of the three freshly spiked soils S1 to S3 was 103% considering all three spike levels (0.5, 2.5, and 10 ng/g). In total, 97% of all determined relative recoveries were within the range of 70–120% (see Table 2; for individual relative recoveries see ESM-A Table S10). As described in ESM-B 9, analyte MIX solution was spiked onto the soil 1 h before starting the extraction and the organic solvent was allowed to evaporate. In a preliminary experiment, the time between analyte MIX addition and extraction was extended to 16 h and no differences in relative recoveries were observed compared to an exposure time of 1 h.

For external quality assurance, our laboratory participated in the ring trial *PT-PAS-II.* Two agricultural soil samples (a, b) with native pesticide residues of soil type luvisol from the Czech Republic (tested for their representativeness by the organizer) as well as one blank soil material were shipped to the 24 participating laboratories. In total, 104 pesticides were on the target list of the ring trial, of which 58 were included in the here presented analytical method. *Z*-scores were calculated based on two different algorithms, i.e., ISO 13528 [52] and Horn procedure, of which the latter was used for a small number (4–7) of submitted concentrations per pesticide by the participating laboratories. With median *z*-scores of 1.05 and 1.12 (see Table 2) and *z*-scores < 2 (criteria: 0 ≤ |*z*-score| ≤ 1: good, 1 < |*z*-score| ≤ 2: satisfactory, 2 < |*z*-score| ≤ 3: questionable) for 88% and 93% of the detected pesticides in test soils a and b, respectively, the results of our developed analytical method proofed true. However, only 16 and 14 out of the 29 and 23 detected pesticides in test soils a and b, which overlapped with the target pesticides of the ring trial, respectively, were evaluated with a *z*-score. The reason for this was a too little number of pesticide concentrations submitted by the participating laboratories. All individual *z*-scores for the 16 and 14 evaluated pesticides were positive, i.e., the pesticide concentrations quantified in this study were always higher than the robust average of all submitted pesticide concentrations. Since two soils with native pesticide residues were analyzed and the “true” concentrations were unknown, positive *z*-scores suggest better extraction efficiencies compared to the remaining participating laboratories.

#### Precision

Different precisions (instrumental precisions, intra-day method precisions, inter-day method precisions, and inter-person method precisions) were determined to underline the method performance. Overall, all types of precisions delivered excellent results and precisions were highly comparable no matter whether based on freshly spiked soils, the partly aged soil S2.1, or agricultural field soils. All types of precisions for the individual pesticides are listed in ESM-A Tables S11.1 to S11.4 and summaries as well as the number of replicates analyzed to determine all types of precisions are given in Table 2.

#### Linearity

Matrix-matched calibration curves were linear for all analytes (median *R*^2^ = 0.999 with a minimum *R*^2^ = 0.992) with differing linear ranges for the individual pesticides. The lower limit of the linear range, defined as the concentration of the matrix-matched calibration standard, for which the analyte peak areas of the quantifier and qualifier ion transitions still fulfilled the required S/N and qualifier-to-quantifier ion ratios (see “Method validation”), was on median 0.1 ng/g (minimum/maximum lower limit: 0.05/5 ng/g) and the median upper limit of the linear range was 35 ng/g (minimum/maximum upper limit: 5/50 ng/g) (see Table 2). The lower and upper limits of the linear range for each individual pesticide are listed in ESM-A Table S6.

#### Limits of quantification

With a median ILOQ of 0.025 ng/mL (see Table 2; 0.125 pg/5 µL injection volume), the used LC–MS/MS system is highly sensitive. ILOQs for the individual pesticides are listed in ESM-A Table S6﻿. More relevant are MLOQs, taking into account all steps of the developed method. Those should be substance-specific, as sensitive as possible, and consistent to ensure data comparability among different soils and sites. However, this is challenging due to soil-specific matrix effects during ESI (see “Matrix effects during ESI”). Therefore, matrix-matched calibration using a blank soil matrix largely representative of Swiss agricultural soils (the standard soil S2, see Table 1) was applied and used as a starting point to determine MLOQs. However, S2 can only approximate matrix effects that might occur in different individual field soil extracts. Ideally, matrix effects would have to be determined for every soil matrix to report entirely correct sample-specific MLOQs. Yet, this is not practicable in routine analysis, e.g., for long-term soil monitoring in which consistent MLOQs are pursued to facilitate data evaluation and statistical analysis at trace levels. Therefore, matrix effects for S1 and S3 to S5 using the signal intensities of each analyte in S2 as reference (*case ii*) were determined (see “Method validation”). Based on these findings, it was decided to adjust S2-based MLOQs by a *global matrix correction factor* of two (maximal ion suppression of − 50%), since only three (atrazine-desethyl, atrazine-desisopropyl, and forchlorfenuron) out of the 146 pesticides included in the developed method showed ion suppression higher than − 50% in any of the matrices using S2 as reference (see ESM-B 12 Figure S5 and for individual matrix effects see ESM-A Table S7). This factor is rather conservative since the maximal median ion suppression over all soils was − 24% (S4) and according to the *SANTE 11312/2021* guideline [27], ± 20% matrix effects are considered as not significant. However, when extracting a large number of different soils during a long timeframe, as it is the case in routine monitoring, reliable MLOQs are sought. With a median MLOQ of 0.2 ng/g and ~ 80% of all MLOQs being equal to or smaller than 0.5 ng/g (see Table 2), the developed analytical method is highly sensitive and suitable for soil monitoring (for individual MLOQs see ESM-A Table S6). Moreover, method performance criteria were still fulfilled in this low concentration range (S2 spiked with 0.1 and 0.25 ng/g) and relative recoveries and intra-day method precisions were on median 103% and 3% for both spike levels (individual values are listed in ESM-A Table S10).

The MLOQs of the developed analytical method are between one and two orders of magnitude more sensitive in comparison to other methods dealing with the analysis of pesticides in soil (e.g., MLOQ (number of pesticides included in the respective study) in Geissen et al. [15], 1–20 ng/g (36–75); Silva et al. [8], 10 ng/g (76); Łozowicka et al. [16], 5–10 ng/g (216); Kosubová et al. [9], 3–10 ng/g (64); Hvězdová et al. [10], 3–10 ng/g (68); Colazzo et al. [53], 1–10 ng/g (30)). Only few studies reported MLOQs below 0.5 ng/g (Riedo et al. [6], 0.04–36 ng/g (46); Lafay et al. [11], 0.01–5.5 ng/g (31); Acosta-Dacal et al. [13], 0.5–20 ng/g (218); Homazava et al. [42], 0.1–2.9 ng/g (25); Pose-Juan et al. [12], 0.2–0.7 ng/g (17); Pelosi et al. [18], 0.01–5.5 ng/g (31)), at which most of the studies with MLOQs below 0.5 ng/g analyzed only between 17 and 46 pesticides.

#### Summarized method performance

Overall, the developed analytical method offers the selective and sensitive quantification of 146 pesticides in soil with varying soil properties (soil pH from 3.6 to 7.4 and C_org_ content mostly between 1 and 5%, see Table 1) at trace levels predominantly in the sub-ng/g range. Accurate quantification was ensured by the use of ~ 100 ILIS and their systematic assignment to analytes_nsi-ILIS_ to address the need to analyze many different soils with varying soil properties and thus soil-specific matrix effects, e.g., under routine conditions within long-term soil monitoring. In contrast to the common approach for method validation of soil extraction methods solely based on soil samples spiked with pesticides shortly before the extraction, our method is additionally validated via the partly aged reference soil S2.1 and via agricultural field soils with native pesticide residues. In this way, our figures of merit (see Table 2) such as trueness and precision are supported by soil samples with more realistic binding affinities of pesticides to the soil matrix. Recommendations for quality control under routine conditions are explained in detail in ESM-B 13.

### Application to Swiss (agricultural) soil samples

The developed method was finally applied to eight soil samples from Swiss agricultural fields under conventional agricultural management. These include four cropland sites with varying crop rotations (S5 to S8), one vegetable site (S11), one orchard (S12), and two vineyards (S13 and S14). Additionally, soil samples from two Swiss grassland sites (S15 and S16), one Swiss municipal park (S17), and a “negative control” from the Swiss national park (S18) were analyzed. For details about soil characteristics, see Table 1.

**Fig. 4:**
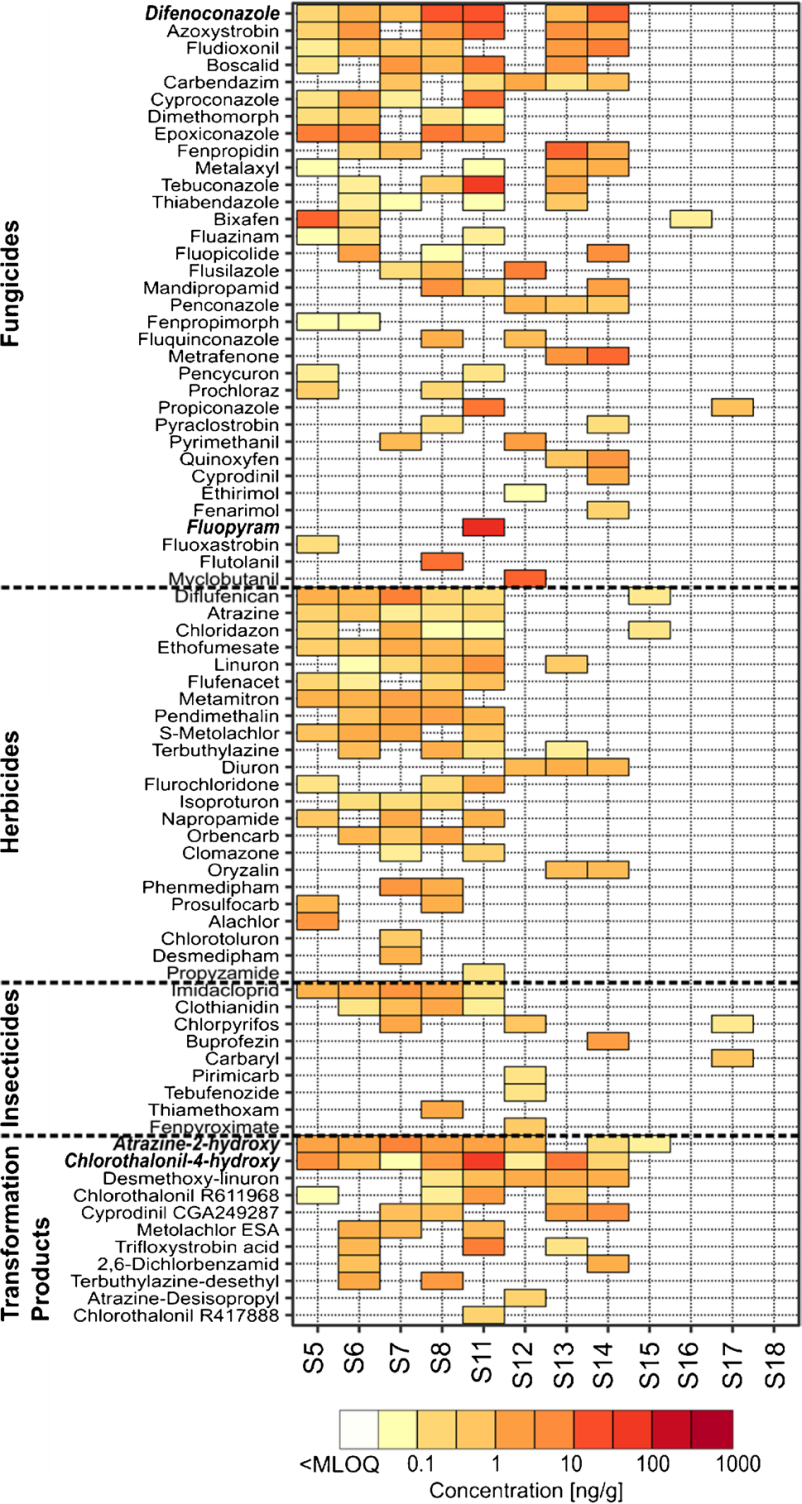
Pesticide concentrations in selected Swiss (agricultural) soils (cropland sites: S5 to S8, vegetable site: S11, orchard: S12, vineyards: S13 and S14, grassland sites: S15 and S16, municipal park: S17, and national park: S18). Only pesticides that exhibited concentrations above MLOQ in at least one soil sample are shown (77 out of 146). Each row represents one pesticide and each column one site. Pesticides are ordered by pesticide class, i.e., fungicides, herbicides, insecticides (including the acaricide fenpyroximate), and transformation products. Within each pesticide class, pesticides are ordered by detection frequency over all sites and then alphabetically. The color range depicts the concentration level and empty white fields mark concentrations below the corresponding MLOQ. The three most frequently detected pesticides (chlorothalonil-4-hydroxy, atrazine-2-hydroxy, and difenoconazole) as well as the pesticide with the highest quantified individual concentration (fluopyram) in the eight agricultural field sites (S5 to S8 and S11 to S14) are highlighted in bold and italic

Figure 4 shows the quantified soil concentrations of the 146 pesticides included in the developed method (individual concentrations are listed in ESM-A Table S12). Altogether, 77 different pesticides were quantified (> MLOQ) over all sites. The highest number of different pesticides was found in the cropland site S8 and in the vegetable site S11 (*n* = 37), followed by the cropland sites S6, S7, and S5 (*n* = 32, *n* = 31, and *n* = 29, respectively), the vineyards S14 and S13 (*n* = 21 and *n* = 22, respectively), the orchard S12 (*n* = 16), the municipal park S17 (*n* = 3), and the grassland sites S15 and S16 (*n* = 3 and *n* = 1, respectively). No pesticides were found in the Swiss national park (S18), which highlights its remoteness and protection status as well as the method performance, i.e., the lack of blank contamination. The higher number of different detected pesticides on the cropland sites compared to the permanent cultures such as vineyards or orchards is in line with our expectations. Crop rotations on the cropland sites lead to the application of a broader pesticide pattern compared to the pesticide applications on permanent cultures, where rather the same pesticides are applied each growing season.

Herbicides were detected more frequently in soils from the cropland sites (S5 to S8) compared to those from vineyards (S13 and S14), where  ~ 75% of the total number of detected pesticides were fungicides or thereof TPs (see Fig. 4). The three most frequently detected pesticides in the eight agricultural field sites (S5 to S8 and S11 to S14) were the TP chlorothalonil-4-hydroxy (8/8 sites) followed by the TP atrazine-2-hydroxy (7/8 sites) and the fungicide difenoconazole (7/8 sites). Chlorothalonil, a broad-spectrum fungicide, has been banned in the European Union and Switzerland in 2019 [54, 55] due to its carcinogenic properties and the detection of potentially toxic chlorothalonil TPs in groundwater. The analyzed agricultural sites were sampled between 2016 and the beginning of 2019, which was before the general ban on chlorothalonil in Switzerland. The phenolic TP chlorothalonil-4-hydroxy, which has been detected in all agricultural sites, exhibits a medium to low mobility (K_fOC_: 250–718 L/kg [56]), and the mobility increases for the second phenolic chlorothalonil TP R611968 (K_fOC_: 51–128 L/kg [56]), which has been detected in 4/8 sites. In contrast, the sulfonic acid TP R417888 is much more mobile (K_fOC_: 5–17 L/kg [56]) and has only been found in 1/8 sites. This is in line with more frequent detections of sulfonic acid chlorothalonil TPs (e.g., R417888) in groundwater samples compared to the phenolic chlorothalonil TPs (e.g., R611968), which are less mobile and therefore retained in soil [57–59]. However, chlorotalonil-4-hydroxy was not analyzed in the before mentioned studies. The frequent occurrence of atrazin-2-hydroxy, a TP of the herbicide atrazine, which has been banned in the European Union in 2005 [60] and in Switzerland in 2009 [34], was already observed in several studies [6, 7, 9, 17, 61] and points towards a legacy of high atrazine application rates in the past. Within the *AP PPP* [19], the fungicide difenoconazole is listed as a pesticide with high-risk exposure due to its persistence (soil degradation in the field, DT_50_: 20–265 d [51]) and toxicity (no effect concentration (NOEC) earthworms: 200 ng/g, NOEC Daphnia magna: 0.0056 mg/L [51]). Its frequent occurrence in agricultural soils in concentrations that get close to the terrestrial NOECs strengthens the decision to list this pesticide as a candidate for substitution.

Fungicides and thereof TPs reached the highest individual pesticide concentrations in the analyzed soils (see Fig. 4). Especially on the vegetable site (S11), the highest individual pesticide concentrations up to 140 ng/g were quantified for fluopyram. However, individual pesticide concentrations in agricultural soils are strongly dependent on the time of sampling, the time of pesticide application, and the respective soil degradation rates (e.g., [12, 62]). Sampling predominantly took place in the wintertime when usually no pesticides are applied, yet the vegetable site (S11) and the orchard (S12) were sampled at the beginning of April.

The median sum concentration of all individual pesticides per agricultural site (S5 to S8 and S11 to S14) was 92 ng/g and the highest sum concentration was found in soil from the vegetable site (S11, 500 ng/g). All 46 pesticides included in the study by Riedo et al. [6] were part of the herein-developed method. When comparing the sum concentrations quantified in each analyzed agricultural soil, either based on the subset of 46 pesticides or based on all 146 pesticides included in the developed method, a median percentage increase in the sum concentrations of 48% was observed when considering all 146 pesticides. Correspondingly, the median percentage increase in the number of detects per agricultural site was 47%. This distinct percentage increase in sum concentration and number of detects confirms our target analyte selection and its relevance for long-term soil monitoring. The here presented multi-residue method for trace analysis of 146 pesticides in soil will now be applied to soil samples from various monitoring campaigns within the *AP PPP* [19], to provide a terrestrial exposure assessment as a basis to review one of its overarching goals that pesticide applications have no long-term negative effects on soil fertility.

### Supplementary Information

Below is the link to the electronic supplementary material.

Supplementary file1 (XLSX 589 KB)

Supplementary file2 (DOCX 8887 KB)
