# Supplementary material for: A multi-residue method for trace analysis of pesticides in soils with special emphasis on rigorous quality control
Source: Anal Bioanal Chem. 2023 Aug 8;415(24):6009–25. doi: 10.1007/s00216-023-04872-8 (PMC10556155; doi:10.1007/s00216-023-04872-8)
Supplement: Supplementary file 2 — Supplementary file2 (DOCX 8887 KB) [file 216_2023_4872_MOESM2_ESM.docx]

**Electronic Supplementary Material B (ESM-B) to**

**A multi-residue method for trace analysis of pesticides in soils with special emphasis on rigorous quality control**

Andrea Rösch^‡^, Felix E. Wettstein^‡^, Daniel Wächter^§^, Vanessa Reininger^§^,
Reto G. Meuli^§^, Thomas D. Bucheli^‡*^

^‡^ Environmental Analytics, Agroscope, 8046 Zurich, Switzerland

^§^ Soil Quality and Soil Use, Agroscope, 8046 Zurich, Switzerland

*Corresponding Author: phone: +41 58 468 73 42

e-mail: [thomas.bucheli@agroscope.admin.ch](mailto:thomas.bucheli@agroscope.admin.ch)

**Table of content**

[ESM-B 1: Analyte MIX solutions 3](#_Toc139659085)

[ESM-B 2: Pesticide selection 3](#_Toc139659086)

[ESM-B 3: Soil samples 4](#_Toc139659087)

[ESM-B 4: Accelerated solvent extraction 5](#_Toc139659088)

[ESM-B 5: Matrix-matched calibration 6](#_Toc139659089)

[ESM-B 6: ILIS selection for analytes without structure-identical ILIS (analytes_nsi-ILIS_) 7](#_Toc139659090)

[ESM-B 7: Analyte selectivity: qualifier-to-quantifier ion ratios 10](#_Toc139659091)

[ESM-B 8: Determination of absolute recoveries_QuEChERS_ 11](#_Toc139659092)

[ESM-B 9: Determination of relative recoveries 11](#_Toc139659093)

[ESM-B 10: Determination of different precisions 12](#_Toc139659094)

[ESM-B 11: LC-ESI-MS/MS optimization 13](#_Toc139659095)

[ESM-B 12: Matrix effects 17](#_Toc139659096)

[ESM-B 13: Recommendations for quality control under routine conditions 19](#_Toc139659097)

[References 20](#_Toc139659098)

## ESM-B 1: Analyte MIX solutions

Individual reference standard solutions were prepared gravimetrically for all analytes and ILIS at 1 mg/mL. The solvents were chosen based on the solubility of the individual analytes and ILIS in organic solvents [1, 2] and the used solvent was mostly acetonitrile (all individually used organic solvents are listed in ESM-A Table S1.1).

An in-house made analyte MIX solution of exact concentration (250 ng/mL) composed of the 146 target analytes was prepared gravimetrically in acetonitrile, which was further diluted to exact concentrations of 25 and 2.5 ng/mL. Additionally, a custom-made analyte MIX solution that contained 137 of the 146 target analytes was ordered at *LGC Standards Ltd*. (Teddington, UK) and the nine missing target analytes were added. It was used as external reference standard solution for method development and validation, and, after transition to routine monitoring, used for quantification. As a second external reference standard solution, a pesticide MIX solution prepared by the *Laboratory of the Canton of Zurich* [3] was used that contained 87 of the 146 target analytes. The three different analyte MIX solutions did not differ systematically (median deviation of 3% based on 5 ng/mL matrix-matched calibration standards, individual data not shown).

## ESM-B 2: Pesticide selection

Pesticides relevant for a long-term soil monitoring were selected based on three categories: (i) their application, (ii) their environmental behavior and (iii) their ecotoxicity. Points were assigned per category (i-iii) if specific criteria were fulfilled (1 point: criteria fulfilled, 0 point: criteria not fulfilled, 0.5 point: no information), that are: (i) the pesticide is among the 20 most frequently applied pesticides or among the 20 quantitatively most used pesticides in Switzerland; for this reason, the top 20 candidates for frequency or amount of used pesticide were assigned separately for each type of crop (cropland, vineyard, orchard and vegetable) to take into account the differently sized areas per crop type, (ii) soil degradation measured in terms of half-lives (DT_50_) (DT_50, lab_ at 20 °C in the laboratory with a pF value of two reflecting the soil moisture ≥ 60 days) and mobility measured in terms of organic carbon-water partition coefficients (K_OC_) or organic-carbon normalized Freundlich distribution coefficients (K_fOC_) (K_(f)OC_ ≥ 500 L/kg) and (iii) acute or chronic toxicity for the most sensitive soil organism (*half-maximal lethal concentration* (LC_50_), *half-maximal effective concentration* (EC_50_) or *half-maximal effective rate* (ER_50_) ≤ 10 mg/kg; *no-observed-effect concentration* (NOEC) ≤ 1 mg/kg) and bioaccumulation potential measured in terms of octanol-water partition coefficients (log K_OW_) (log K_OW_ ≥ 4). Finally, the sum of all assigned points per category was averaged and a pesticide or thereof transformation product was rated as relevant if the “average score” was ≥ 0.5 (scores per category and the average score for each selected pesticide together with the major substance characteristics (DT_50_, K_(f)OC_, log K_OW_ and NOECs for the most sensitive species) are listed in ESM-A Table S2).

## ESM-B 3: Soil samples

The LUFA (S1 and S2) and the WEPAL (S3 and S4) samples represent topsoil (0-20 cm). After reception, the LUFA soils were dried (at 40 °C until constant weight (48 h)) and sieved (< 2mm). The WEPAL soils were used as received (dried at 40 °C until constant weight (48 h), milled and sieved < 0.5 mm).

Swiss agricultural field soils (S5 to S16) and the “negative controls” (S17 and S18) were collected between 2015 and 2019 from long-term monitoring sites throughout Switzerland, which are run by the Swiss Soil Monitoring Network (NABO). Soils were sampled and further processed as described by Gubler et al. [4]. In brief, for each site four replicate samples were taken from the topsoil (0-20 cm). Each replicate consisted of 25 subsamples, taken from an area of 10 m by 10 m using a stratified random sampling design. Soil samples were then subjected to 40 °C until constant weight (48 h), sieved < 2 mm to remove coarse material such as plant debris and were finally stored in high-density polyethylene containers in the dark at temperatures between 12-15 °C. Additionally, four soils (S5 to S8) were directly frozen after sampling
(-20 °C), slightly defrosted in the fridge before sieving < 5 mm using liquid nitrogen, split to generate subsamples accessible for individual analysis and each subsample was finally stored in aluminum containers in the dark at -20 °C (called “undried soil samples”).

## ESM-B 4: Accelerated solvent extraction

Method performance criteria such as extraction efficiencies and intra-day method precisions of the developed QuEChERS method were compared with those of a previously applied and slightly adapted accelerated solvent extraction (ASE) method (Dionex ASE 350, *Thermo Scientific*, Waltham, USA) developed in our laboratory [5].

Each 5 g soil were weighed into 11 mL stainless steel extraction cells. The cells were prefilled with ~1 g of sea sand and were covered with another 1 g of sea sand. At the bottom and the top of the cell a glass fiber filter (Whatman GF/F) was added. Table S1 displays the extraction parameters used during ASE.

Table S1: Extraction parameters used for accelerated solvent extraction.

| Pressure [psi] | 1600 |
| --- | --- |
| Temperature [°C] | 90 |
| Preheat time [min] | 0 |
| Heat time [min] | 5 |
| Static time [min] | 7 |
| Flush volume [%] | 100 |
| Purge time [sec] | 60 |
| Static cycles | 3 |
|  |  |
| Oven off | empty |
| Purge during preheat | empty |
| Bypass heat time | empty |
|  |  |
| **Extracting agent** |  |
| Acetone [% v/v] | 67.5 |
| 1% phosphoric acid in *nanopure*-H_2_O [% v/v] | 15 |
| Acetonitrile [% v/v] | 12.5 |
| Methanol [% v/v] | 5 |

The extraction end volumes of each field soil sample and S2 were adjusted to 30 mL using the extracting agent. Then, 3 mL of each total ASE extract was transferred to conical glass vials, ILIS MIX solution was added, the extracts were evaporated under a gentle stream of nitrogen to dryness and were reconstituted in acetonitrile/*nanopure*-H_2_O 30:70 [% v/v]. S2 matrix was used to prepare a matrix-matched calibration curve. To this end, each 3 mL S2 extract, ILIS MIX solution and analyte MIX solution at respectively complementary volumes were added and the extracts were evaporated and reconstituted as described above.

For this comparison, QuEChERS extracts were treated in the same way, i.e., were evaporated under a gentle stream of nitrogen to dryness and were reconstituted in acetonitrile/*nanopure*-H_2_O 30:70 [% v/v]. In the final QuEChERS protocol, evaporation and reconstitution were redundant since 100% v/v acetonitrile (2.5% formic acid) did not impair the chromatographic peak shapes, since only 5 µL extract were injected using a flow rate of 750 µL/min.

## ESM-B 5: Matrix-matched calibration

Matrix-matched calibrations standards with concentrations of 0.05 – 0.1 – 0.25 – 0.5 – 1 – 2.5 – 5 – 10 - 17.5 – 25 – 35 – 50 ng/mL (equivalent to ng/g) were prepared. To this end, LC-vials were filled with between 0.78 mL and 0.96 mL S2 extract, analyte MIX solution at respectively complementary volumes and 0.02 mL ILIS MIX solution (final concentration in each calibration standard 1, 5 or 15 ng/mL), to reach a final volume of 1 mL in each calibration standard. The slightly different volumes of S2 extract in each calibration standard did not lead to different analytical sensitivities due to different matrix effects (median relative standard deviations (RSD) of ILIS peak areas: 4% based on all calibration standards; individual ILIS data not shown). Final extract volume adjustment is neither necessary, nor feasible in routine method application.

## ESM-B 6: ILIS selection for analytes without structure-identical ILIS (analytes_nsi-ILIS_)

To select ILIS for analytes_nsi-ILIS_, the raw data from the relative recovery experiment (see chapter “2.7. Method validation” in the main text of this publication) was used, in which the five soils S1 to S5 were analyzed spiked (2.5 ng/g) and unspiked. Then, peak area ratios for each analyte_nsi-ILIS_ were built for all possible analyte_nsi-ILIS_ – ILIS combinations in all tested soil types using a script (https://github.com/dutchjes/TFAnalyzeR/blob/master/RelativeRecoveryCalculation.R) and applying the following criteria:

1. For analytes_nsi-ILIS_ that ionize in ESI+ and ESI-, respectively, only ILIS of the same ionization mode were considered.
2. For analytes_nsi-ILIS_ that ionize in ESI+ (n=40), only ILIS within a retention time window of ±2 min around the retention time of each analyte were considered. No retention time restrictions were set for analytes_nsi-ILIS_ that ionize in ESI- (n=11) since in total only eight ILIS are contained in the method that ionize in ESI-.

In a next step, analyte_nsi-ILIS_ – ILIS combinations were selected that ideally led to relative recoveries between 70 and 120% [6] in all five tested soils. If different analyte_nsi-ILIS_ – ILIS combinations led to relative recoveries in the requested range, an ILIS with the closest retention time and with structural similarity to the corresponding analyte (*e.g.*, the combination picoxystrobin-azoxystrobin-d4) was given preference.

The following figures (Figure S1 and S2) are discussed in the main text of this publication.


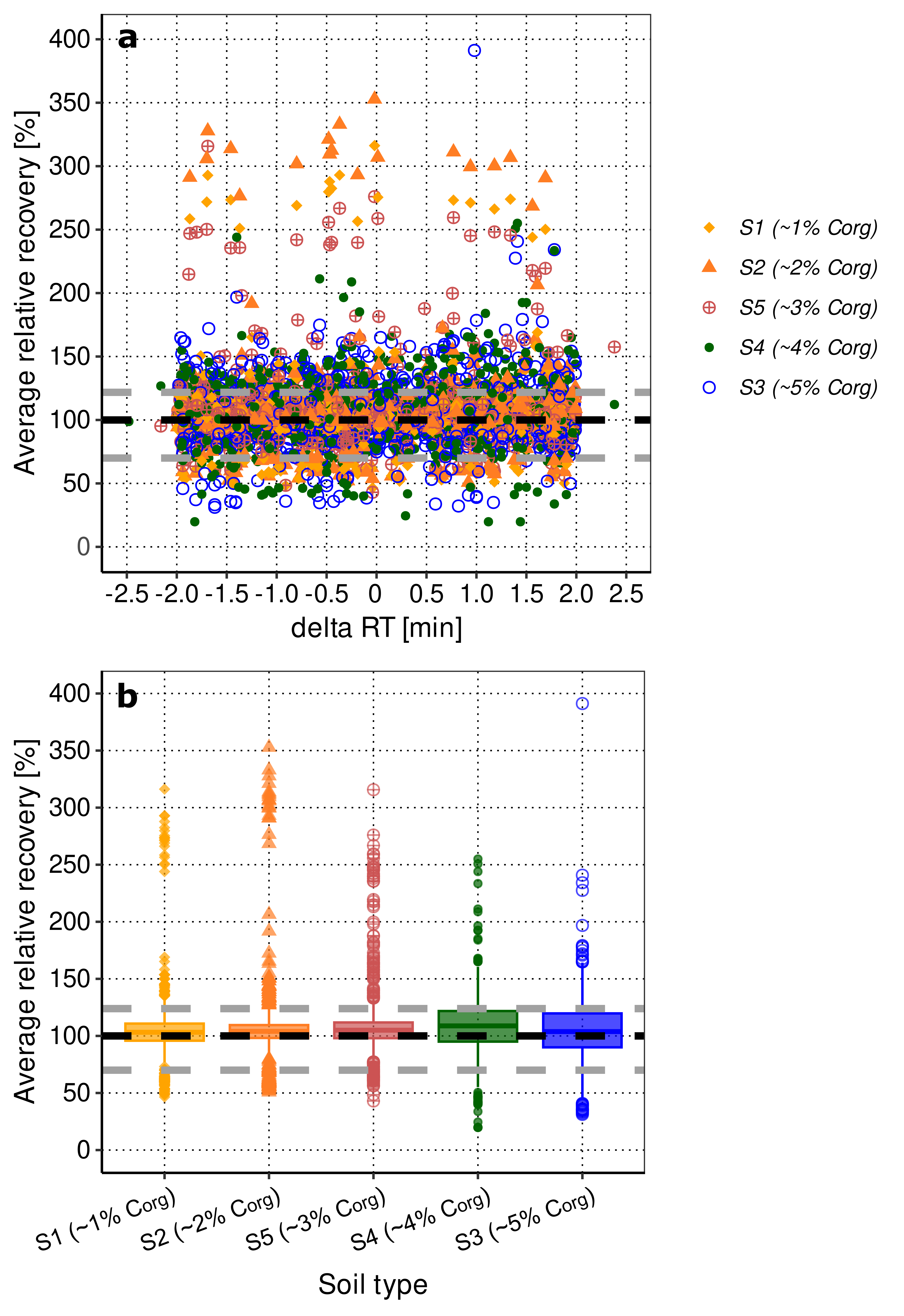


Figure S1: Relative recoveries for all possible analyte (ESI+) - ILIS combinations for analytes_nsi-ILIS_ (analytes without structure-identical-ILIS) based on the applied criteria (see text). Panel a displays the mean relative recoveries (quadruplicate sample preparations for S1 to S5) for S1 to S5 plotted against the retention time difference (delta RT [min]) of each analyte to the selected ILIS. Panel b displays the same data as boxplots individually for S1 to S5. The black dotted lines marks 100% relative recovery, whereas the grey dotted lines mark the requested range of relative recoveries (70-120%).


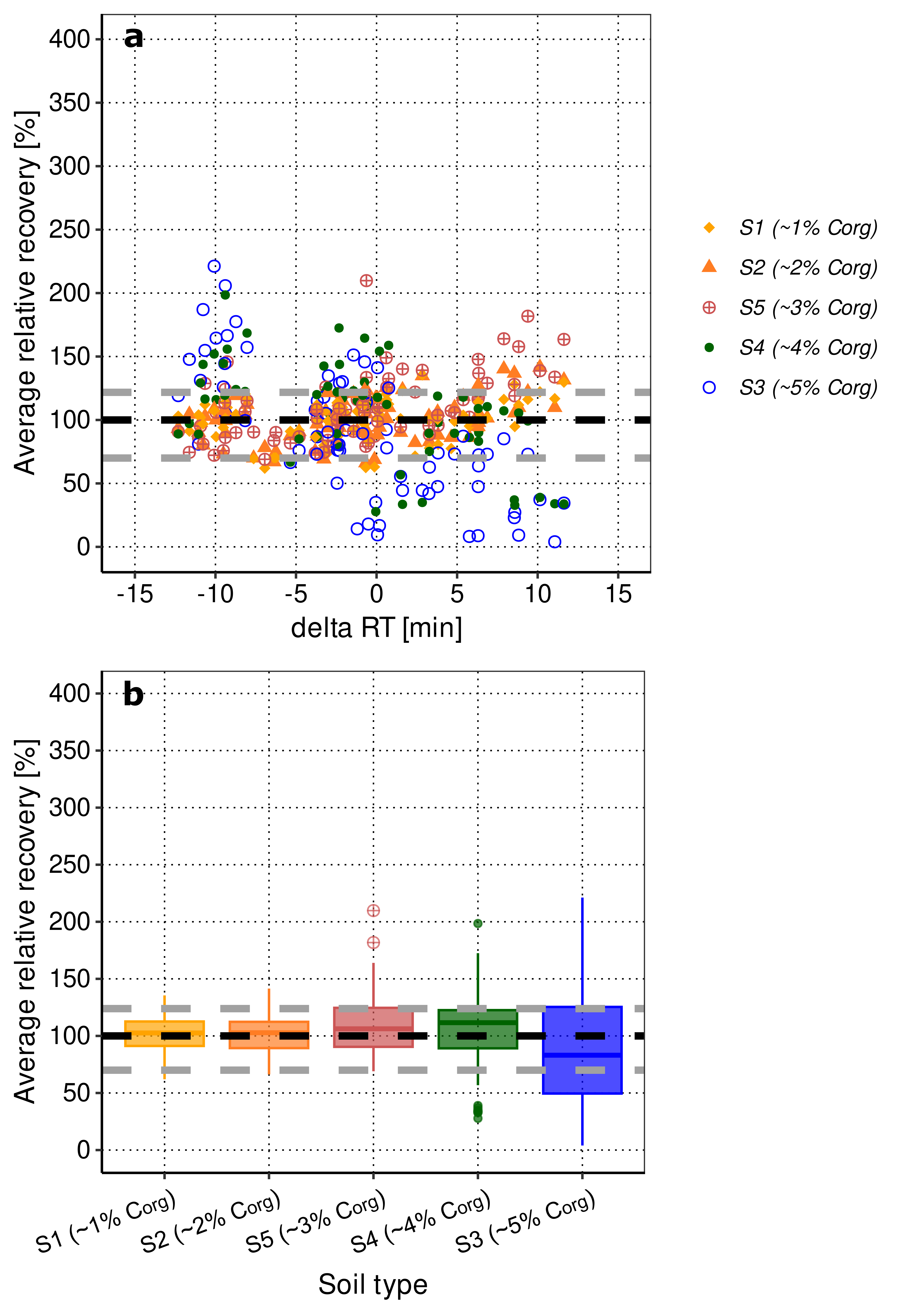


Figure S2: Relative recoveries for all possible analyte (ESI-) - ILIS combinations for analytes_nsi-ILIS_ (analytes without structure-identical-ILIS) based on the applied criteria (see text). Panel a displays the mean relative recoveries (quadruplicate sample preparations for S1 to S5) for S1 to S5 plotted against the retention time difference (delta RT [min]) of each analyte to the selected ILIS. Panel b displays the same data as boxplots individually for S1 to S5. The black dotted lines marks 100% relative recovery, whereas the grey dotted lines mark the requested range of relative recoveries (70-120%).

## ESM-B 7: Analyte selectivity: qualifier-to-quantifier ion ratios

Qualifier-to-quantifier ion ratios (peak area of the qualifier ion transition divided by the peak area of the quantifier ion transition) of each analyte in the soil samples were allowed to deviate from the corresponding average qualifier-to-quantifier ion ratio of the matrix-matched calibration standards according to the criteria defined in the EC Directive 2002/657/EC [7] and listed in Table S2.

Table S2: Allowed deviation of the qualifier-to-quantifier ion ratio of each analyte in a soil sample compared to the average qualifier-to-quantifier ion ratio of the analyte in matrix-matched calibration standards.

| **Relative intensity of the qualifier to the quantifier ion transition (% of the base peak)** | **Allowed deviation for LC-MS applications** |
| --- | --- |
| > 50% | ± 20% |
| > 20% - 50% | ± 25% |
| > 10% - 20% | ± 30% |
| ≤ 10% | ± 50% |

## ESM-B 8: Determination of absolute recoveries_QuEChERS_

To determine **absolute recoveries_QuEChERS_** based on S1 to S3 (organic carbon (C_org_) content: ~1, 2 and 5%, see Table 1 in the main text of this publication) three different treatments were analyzed: (i) the soils were spiked with analyte MIX solution to a concentration of 2.5 ng/g hour (final extract concentration 2.5 ng/mL) before the extraction (the organic solvent of the spiked analyte MIX solution was allowed to evaporate during at least one) and ILIS MIX solution was added after the extraction, (ii) the soils were extracted unspiked (as received) and analyte (final extract concentration 2.5 ng/mL) and ILIS MIX solution were added after the extraction and (iii) the soils were extracted unspiked and ILIS MIX solution was added after the extraction to account for background contamination, i.e., the presence of native pesticides. All samples were processed in quadruplicate and were extracted and analyzed as described in chapters “2.4. Soil extraction” and “2.5. Chemical analysis using LC-ESI-MS/MS” in the main text of this publication. Absolute recoveries were calculated as follows:

$${Absolute recoveries}_{\mathrm{QuEChERS}}= \frac{{\overline{\mathrm{PAR}}}_{\left( i \right) analyte MIX \left( b.E. \right), ILIS MIX (a.E.)}-{\overline{\mathrm{PAR}}}_{\left( \mathrm{iii} \right) no analyte, ILIS (a.E.)}}{{\overline{\mathrm{PAR}}}_{\left( \mathrm{ii} \right) analyte MIX \left( a.E. \right), ILIS MIX (a.E.)}- {\overline{\mathrm{PAR}}}_{\left( \mathrm{iii} \right) no analyte, ILIS (a.E.)}}$$

***equation S1***

where $\overline{PAR}$ is the average PAR and b.E. and a.E. stand for the addition of MIX solution before and after the extraction, respectively. “No analyte” indicates that no analytes were added.

## ESM-B 9: Determination of relative recoveries

To determine **relative recoveries**, S1 to S3 were spiked with analyte MIX solution to concentrations of 0.5, 2.5 and 10 ng/g before the extraction. Spike levels were chosen to cover concentrations close to MLOQ for a majority of the analytes (for MLOQs see chapter 3.2., section “Limits of quantification” in the main text of this publication), ~5 x MLOQ and ~20 x MLOQ. Samples were processed in quadruplicate. To determine relative recoveries directly at the MLOQ for the most sensitive substances, S2 was spiked with analyte MIX solution to concentrations of 0.1 and 0.25 ng/g. Each spike level was analyzed in duplicate. Additionally, relative recoveries were determined for S4 and S5 (spike level 2.5 ng/g) in quadruplicate. Relative recoveries (spike level 2.5 ng/g) of S1 to S5 were used to select ILIS for analytes_nsi-ILIS_ (see chapter 3.1., section “ILIS selection for analytes without structure-identical ILIS” in the main text of this publication). In all these cases, analyte MIX solution was spiked directly onto the soils and the organic solvent of the spiked analyte MIX solution was allowed to evaporate during at least one hour. S1 to S5 were additionally extracted unspiked (as received) in duplicate, to account for potential background contamination, i.e., native pesticide residues. All samples were spiked with ILIS MIX solution before the extraction and were extracted, analyzed and quantified as described in chapters “2.4. Soil extraction”, “2.5. Chemical analysis using LC-ESI-MS/MS” and “2.6. Quantification” in the main text of this publication.

## ESM-B 10: Determination of different precisions

**Instrumental precisions** were estimated via quintuplicate sample injections of a 0.5 and 5 ng/mL matrix-matched calibration standard prepared with the unspiked S2 extract. **Intra-day method precisions** were calculated for all target analytes based on the relative standard deviation (RSD) of the spiked soil samples S1 to S3 utilized for the determination of relative recoveries (quadruplicate samples analyzed in parallel). Additionally, intra-day precisions were calculated via four real agricultural soil samples (S5 to S8, quadruplicate samples analyzed in parallel), in which analytes were subject to environmental sequestration processes. **Inter-day method precisions** were determined for all target analytes based on S2.1 (16 independent sample preparations in duplicate within six months) and additionally via the four real agricultural soil samples S5 to S8 (two sample preparations in quadruplicate within three months). **Inter-person** **method precision** was calculated based on S2.1 (person 1: nine independent sample preparations in duplicate within six months, person 2: seven independent sample preparations in duplicate within four months).

## ESM-B 11: LC-ESI-MS/MS optimization

First, for each analyte and isotopically labeled internal standard (ILIS), up to five ion transitions with corresponding collision energies and potentials (declustering, entrance and collision cell exit) were optimized by direct infusion of small MIX solutions containing between 5 and 10 analytes or ILIS (10 ng/mL in *nanopure*-H_2_O:methanol, 1:1, (% v/v), both containing 5 mM NH_4_COOH) using the ion transition optimization routine of the tandem mass spectrometer (MS/MS). In a next step, aliquots of each small MIX solutions were evaporated under a gentle nitrogen stream to dryness and were reconstituted either in *nanopure*-H_2_O:methanol (9:1 (% v/v)) or in S2 extract to concentrations of 2 and 10 ng/mL. Reconstituted MIX solutions and blanks (*nanopure*-H_2_O:methanol (9:1 (% v/v)) and unspiked S2 extract) were measured by liquid chromatography (LC) coupled to MS/MS using a preliminary LC gradient and acquisition methods for each MIX solution containing all five optimized ion transitions, collision energies and potentials per analyte or ILIS. Two ion transitions for each analyte and ILIS were then preselected based on the following criteria: (i) best signal-to-noise ratio in solvent and S2 extract that increased with increasing analyte or ILIS concentration and (ii) absence of signals in both blank samples, leading to in total 484 ion transitions to be included into the final acquisition method.

Second, the interplay of the LC gradient, the detection window, the target cycle time and the respective dwell times was optimized. The overall goal of the chromatographic optimization was to distribute all analytes and ILIS as equally as possible over the chromatographic run. Consequently, fewer ion transitions have to be measured at the same time leading to fewer overlapping ion transitions and thus higher dwell times (amount of time the instrument is collecting data for a specific ion transition) per ion transition. Additionally, the number of overlapping ion transitions is determined by the time window at which each ion transition is monitored around its specific retention time (detection window). Using a LC run time of 29 min (the LC gradient is displayed in ESM-A Table S3.1) and a global detection window of 40 s led to maximum 58 co-occurring ion transitions. Under these conditions, different target cycle times (0.6, 0.7 and 0.8 s) were tested by injecting a MIX solution that contained all analytes and ILIS (1 ng/mL in *nanopure*-H_2_O:methanol (9:1 (% v/v))) in quadruplicate and sensitivities based on peak areas as well as instrumental precisions were compared. Using a cycle time of 0.6 s slightly reduced the dwell times (see Figure S3). Yet, only 5% of all ion transitions exhibited dwell times less than 5 ms (lowest dwell time that can be applied with the used MS/MS: 3 ms; individual dwell times are reported in ESM-A Table S3.3 to S3.5). Additionally, sensitivities as a measure of peak areas were not decreased due to slightly decreased dwell times when using a target cycle time of 0.6 s and were higher for 97% of all measured ion transition with median percentage increases of 4% / 9% compared to using target cycle times of 0.7 s / 0.8 s. Instrumental precisions (see chapter 3.2., section “Precision” in the main text of this publication) did not differ based on different target cycle times and were on median 2%. Overall, the choice of an appropriate target cycle time is a compromise between achieving a sufficient number of scans per peak (required and achieved number of scans per peak ≥10) and preferably high dwell times for each ion transition.

Third, different ion spray voltages (2000, 2500, 3000, 3500, 4500, 5000 and 5500 V) in combination with different ion source temperatures (500, 550, 600, 650 and 700 °C) were tested. However, no combination of the tested ion spray voltages and ion source temperatures pointed towards a unique improvement in signal intensities. Consequently, the parameter combination for electrospray ionization (ESI) used as a starting point (± 3000 V, 550 °C) was maintained.

Fourth, different modifiers (5 mM NH_4_COOH and 5 mM NH_4_COOH in combination with 0.1% formic acid) of the mobile phase (*nanopure*-H_2_O and methanol) were tested to achieve maximal ionization efficiencies. Using 0.1% formic acid in combination with 5 mM ammonium formate did not improve the sensitivities measured in terms of peak areas. Sensitivities were higher for 70% of all ion transitions (median value 12%) using 5 mM ammonium formate alone.

Fifth, to finally review the selectivity of the preselected ion transitions of each analyte, all analytes were injected individually (10 ng/mL in *nanopure*-H_2_O:methanol (9:1 (% v/v)) and were analyzed using the optimized LC-ESI-MS/MS method including all 484 ion transitions. This ensured that no interfering signals appeared across the different acquired ion transitions, i.e., that each analyte only generated signals in the extracted ion chromatograms of its optimized ion transitions. The main reason for interfering signals are isobaric substances (substances that exhibit either the same mass or nearly the same mass) and substances with overlapping isotopic patterns that co-elute from the LC-column and form the same fragments. If non-selective ion transitions were identified for isobaric substances, those were replaced by unique ones. To avoid overlapping isotopic patterns, particularly of bromine and/or chlorine containing analytes and their corresponding structure-identical ILIS, for which only three to four hydrogen atoms are replaced by deuterium, ion transitions of these ILIS were optimized again using a heavier isotopologue as precursor ion. Figure S4 shows the extracted ion chromatograms of all 484 ion transitions included in the final acquisition method and corresponding LC-ESI-MS/MS conditions for all analytes and ILIS are presented in detail in ESM-A Tables 3.2 to 3.5.


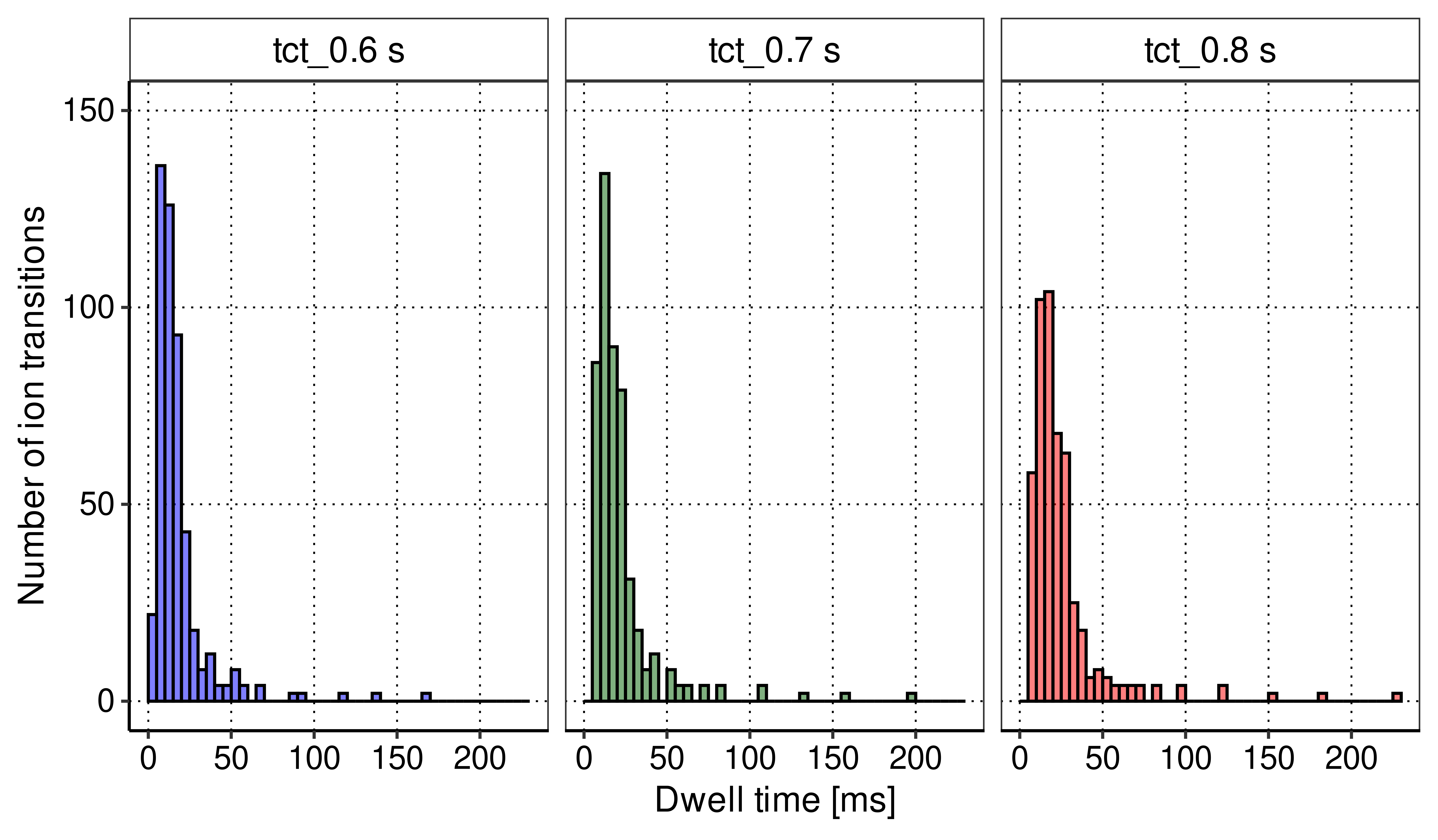


Figure S3: Histograms (bin width: 5 ms) showing number of ion transitions vs. dwell times dependent on different target cycle times (tct) (0.6, 0.7 and 0.8 s) using a global detection window of 40 s.


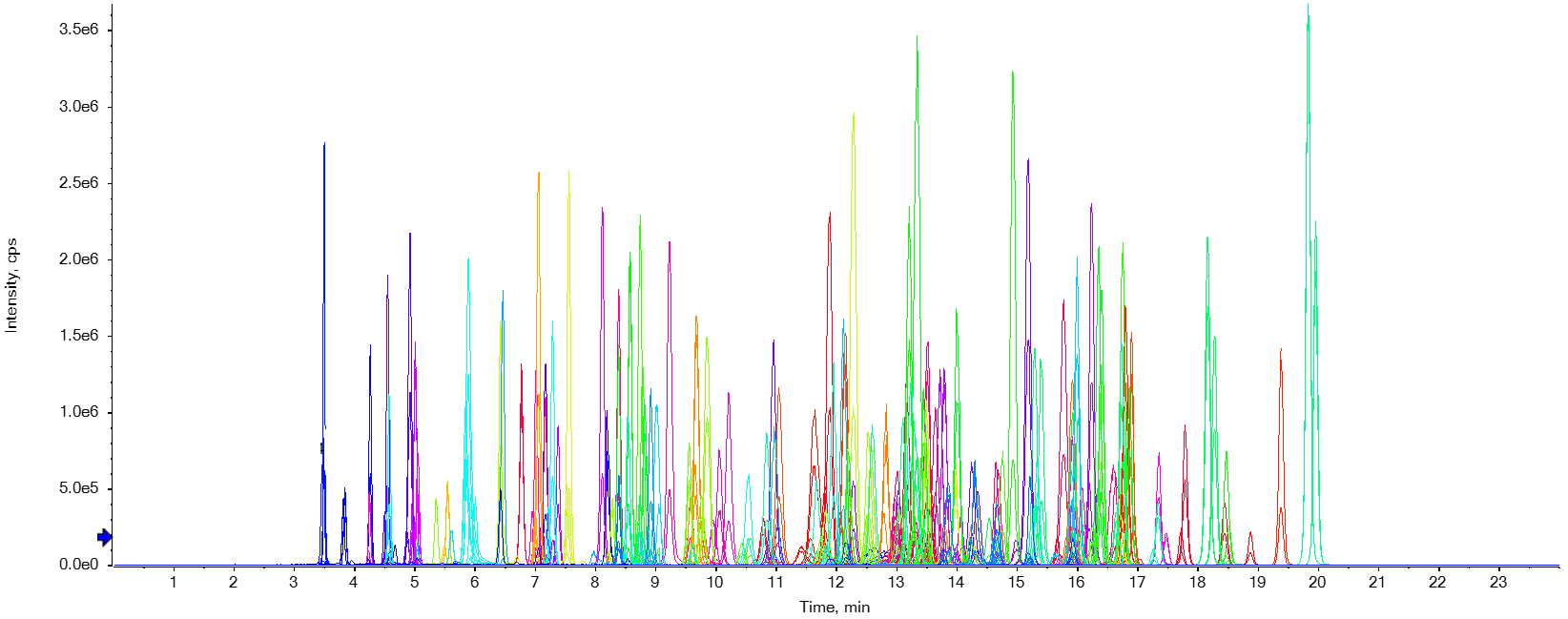


Figure S4: Extracted ion chromatograms (XICs) of all 484 ion transitions of analytes and isotopically labeled internal standards included in the final LC-MS/MS method based on a 5 ng/mL matrix-matched calibration standard.

## ESM-B 12: Matrix effects


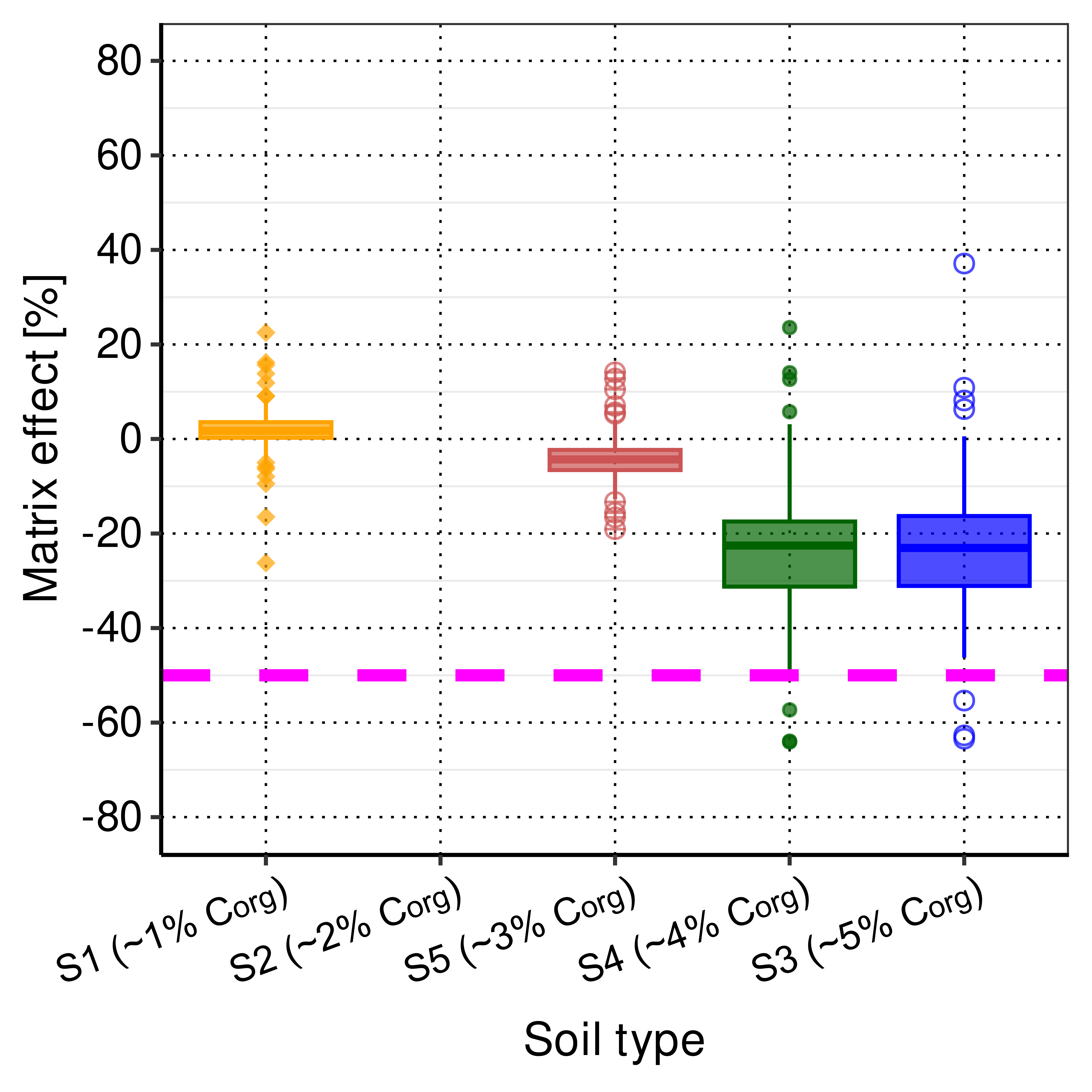


Figure S5: Boxplots of the matrix effects of all pesticides included in the developed analytical method in soils S1 and S3 to S5 with C_org_ contents between 1 and 5% using as reference the signal intensities of each analyte in S2 (C_org_ content of ~2%) extract (*case (ii),* for details see chapter “2.7. Method validation” in the main text of this publication). The pink dashed line marks ion suppression of -50%.


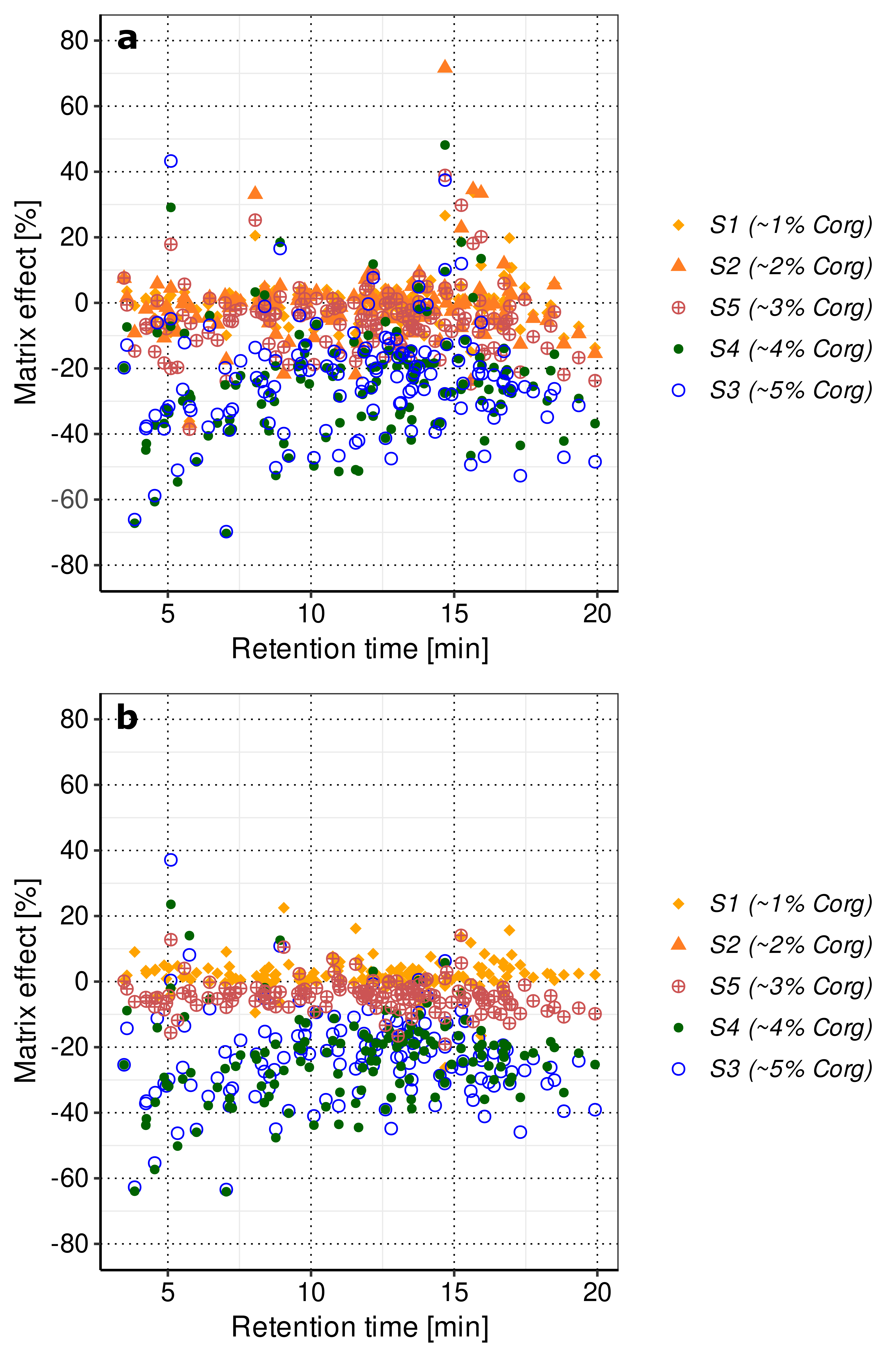


Figure S6: Matrix effects of all pesticides included in the analytical method in soils S1 to S5 with C_org_ contents between 1 and 5% using as reference the signal intensities of each analyte in a calibration standard prepared in acetonitrile (2.5% formic acid) (panel a, *case (i)*) or the signal intensities of each analyte in S2 extract (panel b, *case (ii),* for details see chapter “2.7. Method validation” in the main text of this publication). The matrix effect of each analyte is plotted against the corresponding retention time.

## ESM-B 13: Recommendations for quality control under routine conditions

For quality control under routine conditions, it is recommended to include in every batch of soil samples: (i) a soil with comparable soil characteristics as the routine soils (e.g. S2) analyzed in duplicate, which contains no or a negligible amount of target pesticides and which is used for matrix-matched calibration; those samples should be analyzed once spiked with pesticides before the extraction and once unspiked (used as method blank); in this way, (1) trueness is determined in terms of relative recoveries based on freshly spiked soil samples and (2), by comparing ILIS peak areas in the extracts of S2 with those in the extracts of routine soil samples, the suitability of the *global matrix correction factor* of two, (i.e., maximal ion suppression of -50% using S2 as reference), which is used to adjust S2 based MLOQs, is reevaluated regularly, at least for analytes_si-ILIS_, (ii) a (partly-) aged soil (e.g., S2.1), again with similar soil characteristics compared to the routine soils, which preferably contains (spiked) pesticide residues of the majority of all target analytes; after multiple analyses of S2.1 and provided that pesticide concentrations remain stable under appropriate storing conditions, averaged individual pesticide concentrations in S2.1 can be used as target concentrations to determine trueness, (iii) a routine soil sample analyzed in duplicate (spiked with pesticides before the extraction and unspiked), to randomly review the ILIS selection for analytes_nsi-ILIS_ based on relative recoveries and finally (iv) external reference standards to check the overall trueness and the stability of the used analyte MIX solution, respectively.

## References

1. University of Hertfordshire: Pesticide Properties DataBase (PPDB), <https://sitem.herts.ac.uk/aeru/ppdb/en/index.htm>, (last accessed March 2023).

2. The pesticide manual : a world compendium. Worthing CR, Walker SB, British Crop Protection C, editors. Thornton Heath: British Crop Protection Council; 1987.

3. Kantonales Labor Zürich, <https://www.zh.ch/de/gesundheitsdirektion/kantonales-labor.html> (last accessed February 2023).

4. Gubler A, Wächter D, Blum F, Bucheli TD. Remarkably constant PAH concentrations in Swiss soils over the last 30 years. Environ Sci: Process Impacts. 2015;17:1816-28.

5. Riedo J, Wettstein FE, Rösch A, Herzog C, Banerjee S, Büchi L, et al. Widespread Occurrence of Pesticides in Organically Managed Agricultural Soils—the Ghost of a Conventional Agricultural Past? Environ Sci Technol. 2021;55:2919-28.

6. SANTE 11312/2021: Analytical quality control and method validation procedures for pesticide residues analysis in food and feed. 2021.

7. 2002/657/EC: Commission Decision of 12 August 2002 implementing Council Directive 96/23/EC concerning the performance of analytical methods and the interpretation of results.
